# Current perspectives of ubiquitination and SUMOylation in abiotic stress tolerance in plants

**DOI:** 10.3389/fpls.2022.993194

**Published:** 2022-09-20

**Authors:** Madhavi Singh, Ananya Singh, Neelam Yadav, Dinesh Kumar Yadav

**Affiliations:** ^1^Plant Molecular Biology and Genetic Engineering Laboratory, Department of Botany, University of Allahabad, Prayagraj, India; ^2^Department of Botany, University of Allahabad, Prayagraj, India

**Keywords:** 26S proteasome, abiotic stress, deubiquitination, E3 ligases, post-translational modification, SUMOylation, transcription factor, ubiquitination

## Abstract

Post-translational modification (PTM) is a critical and rapid mechanism to regulate all the major cellular processes through the modification of diverse protein substrates. Substrate-specific covalent attachment of ubiquitin and Small Ubiquitin-Like Modifier (SUMO) with the target proteins, known as ubiquitination and SUMOylation, respectively, are crucial PTMs that regulate almost every process in the cell by modulating the stability and fidelity of the proteins. Ubiquitination and SUMOylation play a very significant role to provide tolerance to the plants in adverse environmental conditions by activating/deactivating the pre-existing proteins to a great extent. We reviewed the importance of ubiquitination and SUMOylation in plants, implicating its prospects in various abiotic stress regulations. An exhaustive study of molecular mechanisms of ubiquitination and SUMOylation of plant proteins and their role will contribute to the understanding of physiology underlying mitigation of the abiotic stresses and survival in plants. It will be helpful to strategize the improvement of crops for abiotic stress tolerance.

## Introduction

**Figure 1:**
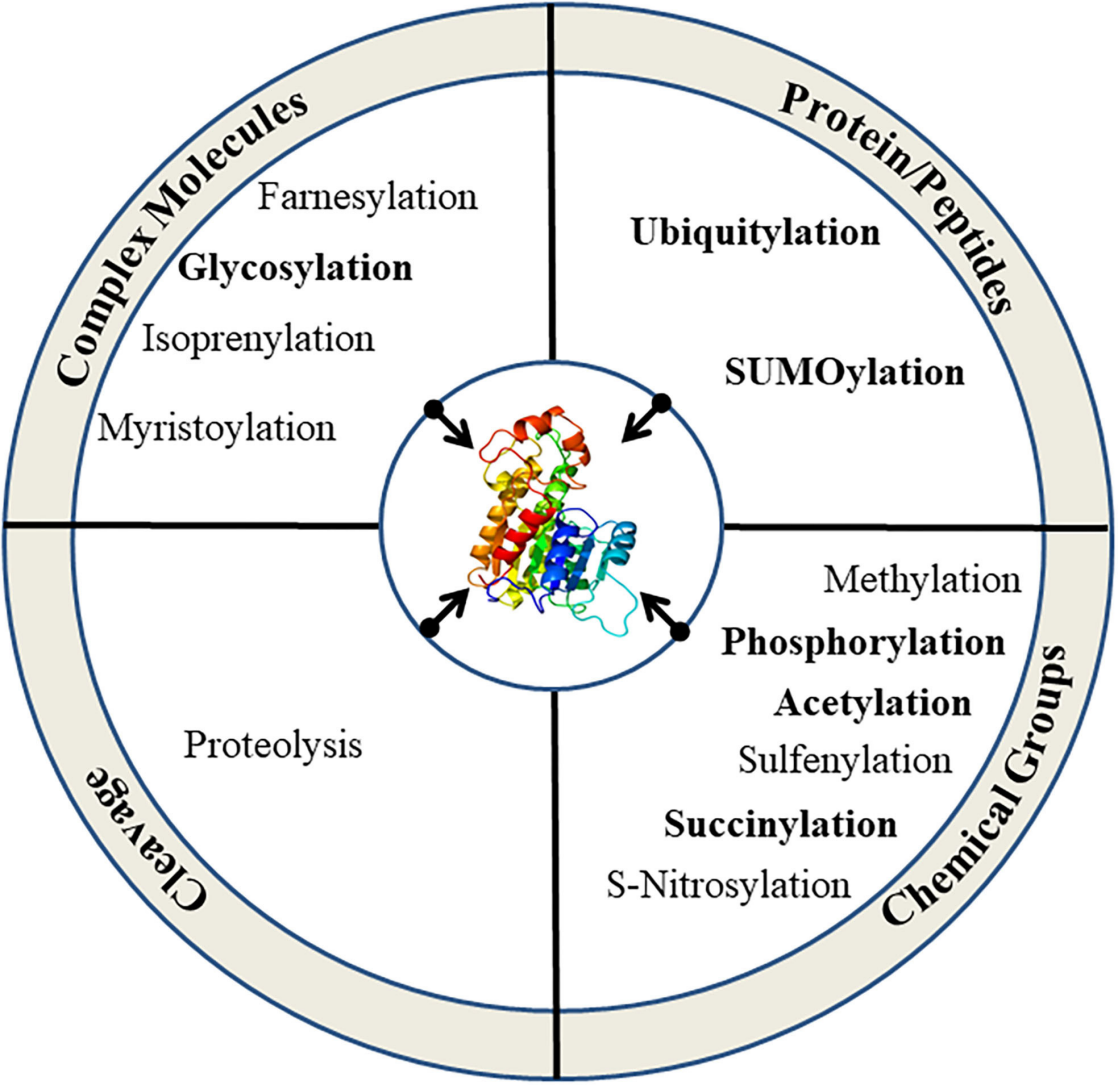
Types of PTMs in eukaryotic cells. More frequent types of PTMs are highlighted.

**Figure 2:**
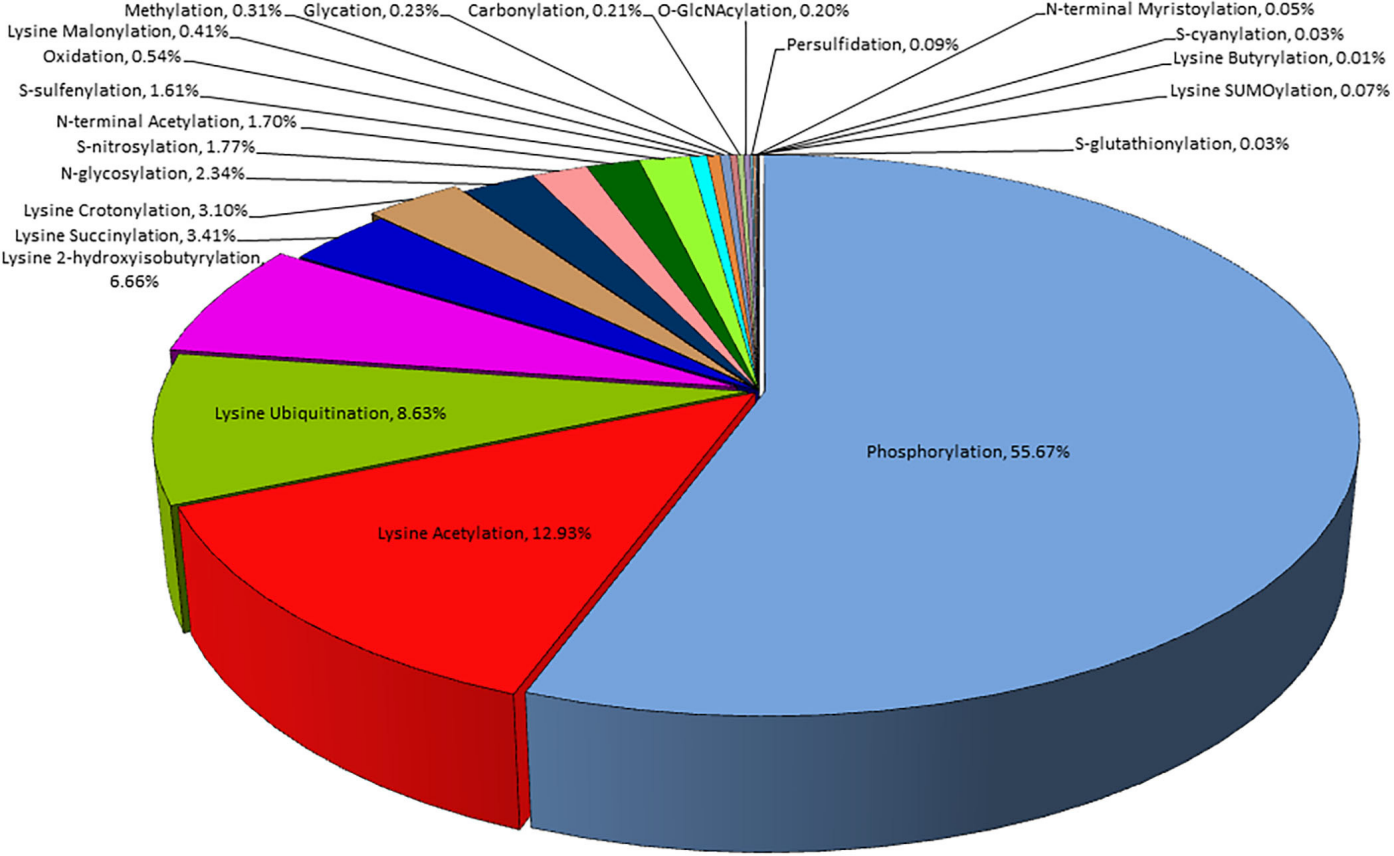
Frequency of common PTM types in plants.

Plants are constantly exposed to a variety of unfavorable conditions which limit their growth. They maintain their homeostasis under stressful environments by modifying several metabolic cascades using complex multi-targeted molecular approaches that eventually alter the proteome to sense and alleviate the effects of stresses (Kosová et al., 2011; dos Reis et al., 2012). Plant proteome is remarkably transformed by post-translational modifications (PTMs) under stressful conditions and plays a key role to alter the structure, function, and abundance of cellular proteins by reversible/irreversible covalent attachment of reactive groups, complex molecules or peptides, and cleavage of oligopeptides (Figure 1; Table 1). PTMs regulate diverse cellular processes, including intracellular and extracellular signal transduction, protein-protein interaction, gene expression, and cell-cell interaction. Thus, PTMs greatly expand the dynamic regulation of cell physiology (Deribe et al., 2010; Shumyantseva et al., 2014). Advancement in proteomic technologies and bioinformatics has immensely facilitated the development of integrative databases of quantitative PTMs in plants (Table 2). Xue et al. (2022), presented >1 million experimentally identified PTM events under 583 conditions for 23 PTM types in 43 plants (http://qptmplants.omicsbio.info; Figure 2). The significance of PTMs in plants can be understood by the fact that 10% of the Arabidopsis genome is dedicated to the two most frequent PTMs viz. phosphorylation and ubiquitination (Lehti-shiu and Shiu, 2012). Furthermore, plants have a larger protein kinase superfamily than other eukaryotes (Mazzucotelli et al., 2006), possibly due to their increased need to be resilient (Hanada et al., 2008). The frequency of PTM types, sites, and chemical moieties covalently attached to proteins in various PTM events are shown in Table 1. Phosphorylation is the most frequent and important PTM-type and plays a crucial role in plant signaling and activation/deactivation of important enzymes/substrates (García-Mauriño et al., 2003), transcription factors (TFs) (Agarwal et al., 2007), and other effector proteins. Histone acetylation regulates gene expression, allowing transcription activation through DNA-binding proteins, and plays a key role under abiotic stresses (Weng et al., 2014; Hu et al., 2015; Li et al., 2021). Recently identified PTM types, such as butyrylation, crotonylation, glutarylation, malonylation, propionylation, succinylation, and 2-hydroxyisobutyrylation, greatly influence the growth, differentiation, and metabolism of plants (Huang et al., 2017; Feng et al., 2021; Xu et al., 2021). Glycosylation has a vital role in protein folding, signaling, intermolecular interactions, cell-cell adhesion, ER signaling, etc. that leads to abiotic stress tolerance. Ubiquitination also plays a critical role in various abiotic stress responses (Ning et al., 2011; Yanagawa and Komatsu, 2012; Peng et al., 2019; Lim et al., 2020; Zhang et al., 2021). SUMOylation is another significant PTM that alters the function, location, and turnover of target proteins and plays a critical role in the development and abiotic stress tolerance (Kurepa et al., 2003; Conti et al., 2008; Castro et al., 2012; Augustine and Vierstra, 2018; Liu et al., 2019; Hu et al., 2022). We reviewed the prospects of molecular mechanism and cross-talk between ubiquitination and SUMOylation of plant proteome under abiotic stress conditions and their role in stress mitigation.

**Table 1:** Frequency of post-translational modification (PTM) types, sites, and chemical moieties covalently attached to proteins in various PTM events.

**PTM-type**	**Frequency of PTM types (%)**	**PTM site on protein**	**Chemical moiety attached**
Phosphorylation	55.67	R-R/K-x-S-ϕ	Phosphoryl- group
Lysine Acetylation	12.93	ε-NH_2_ group of Lysine	Acetyl- group
Lysine Ubiquitination	8.63	ε-NH_2_ group of Lysine	76- residues long Ubiquitin
Lysine 2-hydroxyisobutyrylation	6.66	KxxxxK_hib_; K_hib_xxxxK; KxxxxxxK_hib_; K_hib_xxxxxxK; KTxxxxK_hib_; DxxK_hib_	Hydroxyisobutryl group
Lysine Succinylation	3.41	-NH_2_ group of Lysine	Succinyl- group
Lysine Crotonylation	3.10	ε-NH_2_ group of Lysine	Crotonyl group
N-glycosylation	2.34	-NH_2_ group of Asn in N-X-S/T site	Complex glycans
*S*-nitrosylation	1.77	-SH group of Cys; Phenyl group of Tyr-	Nitric oxide
N-terminal Acetylation	1.70	α-NH_2_ of N-terminus of protein	Acetyl- group
*S*-sulfenylation	1.61	-SH group of Cys (R-SH)	Sulfenic acid (R-SOH)
Oxidation	0.54	Cys/Met/His/Arg/Lys/Pro/Trp	Oxo group,
Lysine Malonylation	0.41	-NH_2_ group of Lysine	Malonyl- group
Methylation	0.31	Lys/Arg/Ala/Asn/His/Asp/Cys/Gly,	Methyl group
Glycation	0.23	ε-NH_2_ group of Lysine/Arginine	Hexoses
Carbonylation	0.21	Lys/Arg/Pro/Thr	Carbonyl group
O-Glycosylation	0.20	-OH group of S/T in -P-(V/T)-g(S)-(S/T)-A	Complex glycans
Persulfidation	0.09	-SH group of Cys, Glutathione disulfide, Protein Sulfenic acid, Cys persulfide	Persulfide (R-S-SH) Polysulfides
Lysine SUMOylation	0.07	ψKXE/D	Small Ub-related protein modifiers
N-terminal Myristoylation	0.05	N-terminal Glycine	Myristoyl- lipid group
*S*-cyanylation	0.03	-SH group of Cys	Cyano group (R-S-CN)
*S*-glutathionylation	0.03	-SH group of Cys	Glutathione group (R-S-SG)
Lysine Butyrylation	0.01	ε-NH_2_ group of Lysine	Butyryl group/Isobutyryl group

**Table 2:** Prediction and analysis tools for ubiquitination sites.

**Tool**	**Algorithm**	**OS**	**Web address**
UBPred	Random Forest	Linux, Windows	http://www.ubpred.org
CKSAAP_UbSite	SVM	Linux	http://protein.cau.edu.cn/cksaap_ubsite/
UbSite	SVM	–	No server
mRMR_Ub Site	Nearest Neighbor	–	No server
UbiNet	Densely connected neural networks	Windows	http://140.138.144.145/~ubinet/index.php
UbiComb	Deep learning	Windows	http://nsclbio.jbnu.ac.kr/tools/UbiComb/
plantsUPS	–	Windows	http://bioinformatics.cau.edu.cn/plantsUPS/

## Ubiquitination

**Figure 3:**
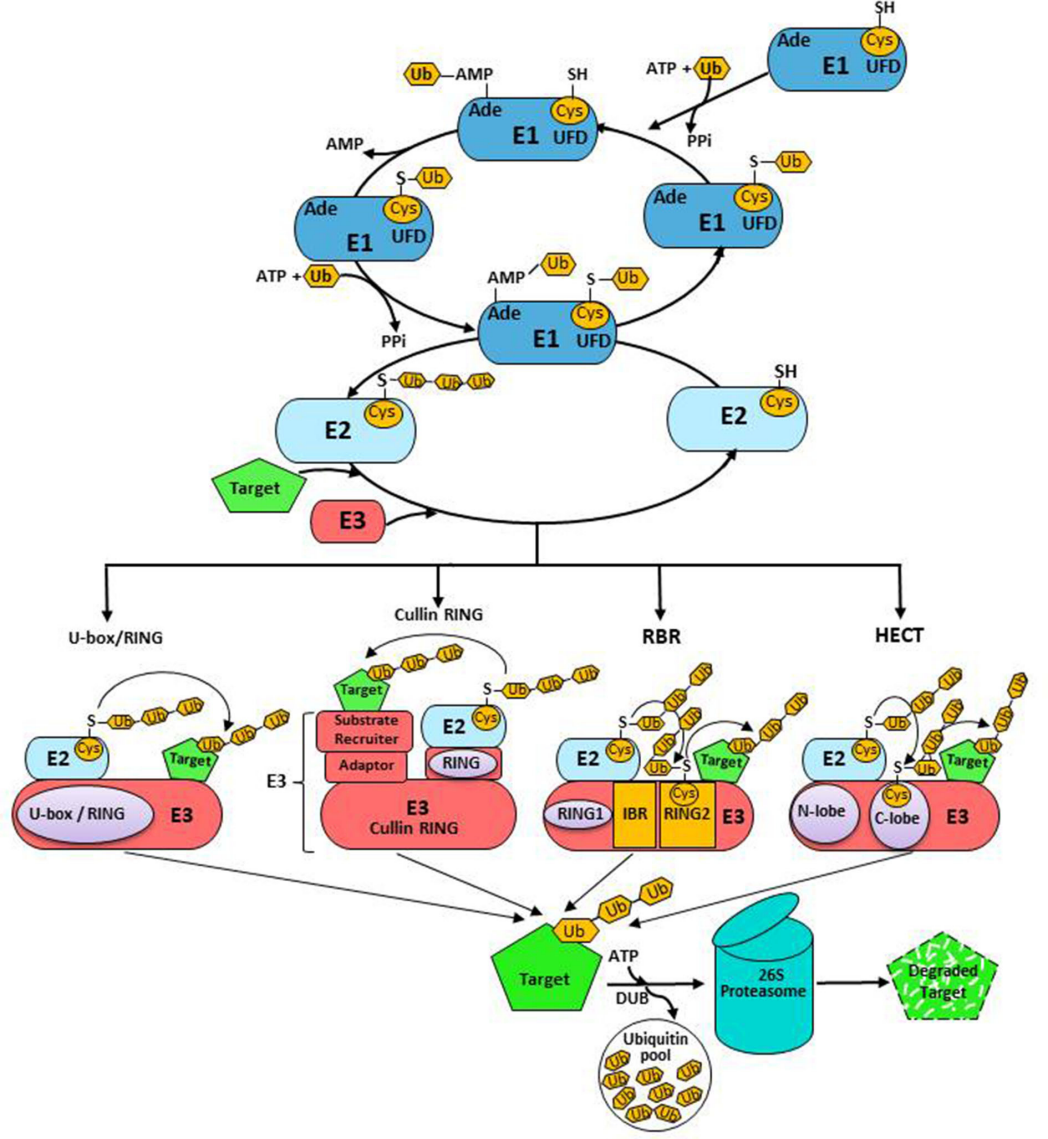
The ubiquitination cascade: Ub conjugation system undergoes ATP-dependent Ub activation and formation of thioester with E1. Subsequently, Ub is transferred to E2. E3 catalyzing Ub transfer to substrates is divided into RING, Cullin RING, HECT, and RBR classes. DUBs catalyze the removal of Ub from substrates.

Protein ubiquitination is the third most frequent, reversible, and extremely diverse PTM type in plants. The ubiquitination processing machinery, Ubiquitin 26S Proteasome System (UPS), plays a crucial role in the precise regulation of gene transcription, DNA replication and repair, quality and abundance of short-lived regulatory proteins, misfolded proteins, and protein trafficking processes in eukaryotes (Vierstra, 2009). The UPS involves covalent attachment of 76 amino acids' long, highly conserved ubiquitin-protein modifier to lysine residues on target proteins in various configurations to accomplish diverse fates (Weissman, 2001). The UPS targets ~80% of the proteins of a eukaryotic cell for degradation (Herrmann et al., 2007). Genome-wide studies showed that ~6% of *Arabidopsis thaliana* genome is committed to UPS, the majority of which code for ubiquitin ligases (E3s) (Yu et al., 2016). The 3-D structure of ubiquitin characteristically has a signature β-grasp domain at the center, enclosing a conserved hydrophobic core. This compact globular structure and extensive H-bonding make ubiquitin unusually stable. Ubiquitin structure displays equipoise between rigidity and flexibility where flexible regions are centered on rigid hotspots to provide high binding affinity and specificity. Ubiquitin protein contains seven highly conserved lysine residues (Lys_6_, Lys_11_, Lys_27_, Lys_29_, Lys_33_, Lys_48_, and Lys_63_), which can be selectively ubiquitinated to produce structurally diverse ubiquitination patterns in the target protein (Kirkpatrick et al., 2006; Kim et al., 2007). The NH_2_- of methionine 1 at the N-terminus also serves as a receptor site for homotypic polyubiquitination (Rittinger and Ikeda, 2017). Diversity in ubiquitination patterns arises from the distinct length and topology of ubiquitin chain(s) attached to specific lysine residues. This interactive ability of ubiquitin might be the reason for the asymmetric complexity of the UPS that reinforces an extended interactome relying on a single molecule. Polyubiquitination of Lys48 of ubiquitin majorly leads to the degradation of misfolded proteins and obsolete proteins, through the ATP-dependent multimeric 26S proteasome system (Mazzucotelli et al., 2008). However, Lys63-linked polyubiquitination of target protein serves as a signal for endocytosis, protein activation, and intracellular trafficking (Pickart and Fushman, 2004). Several ubiquitin moieties attached to the target protein, i.e., monoubiquitination of single or multiple lysine residues, are the additional criteria for ubiquitination and regulate the distribution and function of proteins, thereby regulating various metabolic pathways from transcriptional regulation to membrane transport (Hicke, 2001). Yeast and oat ubiquitin sequences differ by three residues from human ubiquitin but by only two residues from each other. These differences might have led to a functional variation of ubiquitin in plants and animals. However, their major H-bonding patterns and secondary structural features are uniformly conserved. Target proteins are ubiquitinated through sequential reactions carried out by three enzymes: ubiquitin-activating (UBA; E1), ubiquitin conjugation (UBC; E2), and ubiquitin ligase (E3) enzymes. Ubiquitination of protein initiates with the activation of ubiquitin by E1 in an ATP-dependent manner, forming a thioester bond between the C-terminus of activated ubiquitin and the thiol group of a catalytic cysteine residue, forming the E1-Ub intermediate. Thereafter, E2 accepts the activated ubiquitin from the E1-Ub complex and conjugates it onto the cysteine, forming a thioester-linked E2-Ub intermediate (Figure 3). A canonical E1 enzyme has three conserved domains: an adenylation domain for the formation of Ub-adenylate; a catalytic domain with a conserved cysteine residue that links with the di-glycine motif of Ub; and a ubiquitin fold domain (UFD) to associate with E2. In processes where other ubiquitin-like proteins (UBLs) are also part of the conjugation cascade, E1 enzymes confer specificity by matching a particular UBL with only cognate E2s. E2s have 140 amino acid UBC domains, possessing a highly conserved Cys-residue that accepts activated ubiquitin transferred from E1-Ub and facilitates interaction between E3 and E2-Ub intermediate (Wu et al., 2003; Kraft et al., 2005). Although there is an overlap between regions of the UBC domain interacting E1-Ub and E3, the binding of E2 to E1 and E3 is mutually exclusive. Certain members of the E2 family feature variable N- and/or C-terminal extensions that result in functional diversity of E2 to subcellular localization and pathway-specific E1/E2/E3 interactions. E2 enzymes mainly determine the length, specificity of chain assembly, and topology of ubiquitin networks (Kim et al., 2007; Ye and Rape, 2009; Rodrigo-Brenni et al., 2010). E2-E3 combination also determines the topology of the ubiquitin chain (Deshaies and Joazeiro, 2009). E3 ligases are critical regulators that dictate the efficiency and specificity of ubiquitination by recruiting the appropriate target proteins. Simultaneous but asymmetric loading of two ubiquitin molecules on E1, the non-covalent binding of Ub-adenylate, and cysteine-esterification of Ub are remarkable features of the E1-E2 cycle. Studies suggest that coupling of the second adenylation reaction to E2-Ub transfer stabilizes catalytically competent E1 into an energetically favorable conformation, which complements the rate of E2 transthiolation. The E2-Ub complex then concomitantly interacts with group-specific E3 ligases, which, in turn, associate with the target protein to form an isopeptide bond between the C-terminal glycine of activated ubiquitin and Lys of the target protein. This process of ubiquitination is repeated under the strict regulation of E2 and E3 (Komander and Rape, 2012) and can be reversed by deubiquitinating enzymes (DUBs) (Komander et al., 2009). Deubiquitination is the hydrolysis of peptide bonds at highly conserved Gly_76_ residue of ubiquitin, which has constitutive and regulated activities in the cell. DUBs are mainly involved in the following: (i) post-translational processing of ubiquitin precursors that are typically encoded as fusions to itself or other proteins to generate Ub monomers; (ii) protection of activated ubiquitin against various intracellular nucleophiles such as glutathione and polyamines; (iii) regeneration of ubiquitin and/or polyubiquitin chains from Ub-conjugates committed for proteasomal degradation; and (iv) disassembly of unanchored poly-Ub chains to restore and maintain an adequate pool of free ubiquitin in the cell. DUBs can also rescue committed substrates from proteasomal degradation by altering their half-life in response to specific signaling events, thereby serving as the negative regulators of protein degradation. Moreover, DUBs have been proposed to perform final proof-reading activity for the degradation to rescue proteins targeted inappropriately to the proteasome (Lam et al., 1997). DUBs have two signature motifs: the 18 amino acid long Cys-box and a His-box of varying length with conserved catalytic cysteine and histidine residues, respectively. DUBs are crucial in cellular ubiquitin homeostasis and comprise the ubiquitin C-terminal hydrolase (UCH) family and the ubiquitin-specific processing protease (UBP) family. The UCHs preferentially deubiquitinate small peptides and amino acids, while UBPs seem to remove ubiquitin specifically from the proteins targeted for ubiquitination. The Arabidopsis genome expresses 64 DUBs that are classified into five sub-families based on domain organization and catalytic residues: Ubiquitin-specific Protease/Ubiquitin-binding Protease (USP/UBP), Ubiquitin C-Terminal Hydrolase (UCH), Ovarian Tumor (OTU) protease, Machado-Joseph Domain Protease (MJD), and JAB1/MPN/MOV34 (JAMM) protease. The first four are cysteine proteases, while JAMM family members are metalloisopeptidases and require zinc as a cofactor. Often, polyubiquitination and deubiquitination reactions take place in unison, thereby ensuring the abundance of essential enzymes and the regulatory proteins required in the regulatory networks.

The extensive repertoire of E3s differentially modifies diverse target proteins. Plant genomes contain one or two E1s, tens of E2s, and >1,000 of E3s encoding genes (Chen and Hellmann, 2013). The Arabidopsis genome has two E1, 37 E2, 8 E2-like, and >1,300 E3 encoding genes or components of E3 complexes (Stone, 2014). Based on the domain architecture and mechanism of action, the E3 ubiquitin ligases are classified into four classes: Homology to E6-Associated Carboxyl-Terminus (HECT)-type, Really Interesting New Gene (RING)-type/ U-box-type, Cullin RING ubiquitin Ligases (CRLs), and RING-IBR-RING (RBR) (Zheng and Shabek, 2017). Most of the plant E3s belong to the RING-type group and the Arabidopsis genome has >470 genes coding for RING-type E3s (Kraft et al., 2005). The majority of RING-type E3s are monomers containing RING-domain and substrate-recruiting modules in a single polypeptide. However, RING-type domains tend to form homo- or hetero-dimers. Unlike RING E3s that function as allosteric activators, HECT E3s are catalytically active and uniquely form E3-Ub thioester intermediate (Huibregtse et al., 1995) and catalyze polyubiquitination independent of their cognate E2s (Lorenz, 2018). Plant genomes contain significantly higher numbers of U-box protein-related genes as compared to other eukaryotes (Li et al., 2008; Yee and Goring, 2009; Lyzenga and Stone, 2012). The Arabidopsis and rice genomes have 64 and 77 U-box-related genes, respectively. U-box, ~70 amino acid long, is a modification of the RING-finger domain and folds into a scaffold structure like the RING domain. It lacks the classical zinc-chelating cysteine and histidine residues and forms multiple hydrogen bonds using cysteine, serine, and glutamate side chains. Hydrophobic interactions and salt bridges stabilize the corresponding hydrophobic core at the tertiary level (Callis, 2014).

Mechanistically, RBR-type E3s harbor a unique RING1-IN BETWEEN RING (IBR)-RING2 supra-domain that displays defined features of both RING- and HECT-type E3s. The N-terminal RING1, arranged in a cross-brace configuration, represents the typical RING domain, whereas RING2 and IBR domains adopt a common IBR-fold, coordinating two zinc ions that differ from the canonical cross-brace fold of RING1. The C-terminal RING2 is the catalytic subunit of RBR containing a conserved cysteine residue that accepts the donor ubiquitin from E2-Ub conjugate to generate a covalent E3-Ub intermediate, a feature typical of HECT-type E3s. With no catalytic cysteine, the IBR domain possesses two adjacent flexible linkers that enable three RBR sub-domains to adopt multiple conformations relative to each other, which, together with intramolecular interaction with regions outside the RBR domain, allow dynamic autoregulatory transitions between active and inhibited states. As aforementioned, RBR-type E3s function through a concerted RING/HECT-like molecular mechanism, wherein RING1 domains recruit and stabilize the cognate E2-Ub pair, facilitating the transfer of ubiquitin directly onto the RING2 cysteine to form a covalent HECT-like thioester intermediate. Subsequently, this charged Ub couples with its corresponding substrate. Unlike the RING and HECT-type E3s that are involved in the synthesis of diverse homohetrotypic chains, LUBAC, a multi-protein RBR, specifically catalyzes peptide-bond formation between Gly_76_ of existing and Met_1_ of the incoming ubiquitin, yielding linear ubiquitin chains. Most of these ligases further strictly regulate their enzymatic activity by auto-inhibition and activation through specific protein-protein interactions and PTMs (Marín, 2010; Berndsen and Wolberger, 2014; Smit and Sixma, 2014; Dove and Klevit, 2017; Zheng and Shabek, 2017). Forty-two Arabidopsis RBR E3s are categorized into 4 sub-groups: Plant II (22 members), Plant I/helicase (3 members), ARA54 (1 member), and ARIADNE (16 members) (Fernandez et al., 2020). The RBR ligases are reported to regulate the turnover of core components of ABA signaling to modulate plant ABA responses. The ABA receptors, PYR/PYL/RCAR, are ubiquitinated in a spatial- and enzyme-dependent manner for the degradation *via* proteasomal or vacuolar pathways. For example, nuclear ubiquitination of PYR/PYL/RCAR ABA receptors is catalyzed by RFA4 and its cognate E2 UBC26, in addition to known CULLIN-RING E3 ligase (CRL4)-DDB1-ASSOCIATED1 (DDA1) complex, and is degraded in a proteasomal-dependent manner. By contrast, RSL1-mediated ubiquitination of PYL4 and PYR1 at plasma membrane destines modified proteins to endosome-mediated vacuolar degradation pathway through endosomal sorting complex required for transport (ESCRT) (Fernandez et al., 2020).

RING-type E3s exist in different structural contexts, one being multi-protein complexes, where a scaffold protein facilitates the assembly of different components. Plant CRLs are typical examples of multimeric RING E3 ligases and are further classified into four subfamilies: S phase kinase-associated protein 1-Cullin 1-F-box (SCF), Bric-a-brac-Tramtrack-Broad complex (BTB), DNA Damage-Binding domain-containing (DDB), and Anaphase-promoting complex (APC) (Hua and Vierstra, 2011). CRL family of E3 ligases is composed of four or five different protein subunits tethered together through a common adaptor protein. CRL uses Cullin proteins (CUL1, CUL3a/3b, or CUL4) as a platform to interact with E2-Ub binding RING proteins (RBX1/ROC1/ HRT1) at its C-terminal and substrate-recruiting protein at the N-terminal regions (Schwechheimer and Calderon Villalobos, 2004; Hotton and Callis, 2008). The substrate-recruiting subunit can either bind directly to the CUL protein or along with an adaptor protein. Degeneracy between catalytic and adaptor modules allows the association of diverse adaptors to the same catalytic subunit and forms distinct E3 complexes with a broad spectrum of substrate specificity exhibiting enormous plasticity in substrate specificity.

### Ubiquitin code

Attachment of ubiquitin molecules to the substrate proteins in monomeric or polymeric forms linked through specific isopeptide bonds is known as ubiquitin code. Depending upon the linkage type, different ubiquitination patterns attain distinct conformations and lead to unique consequences in cells. Interestingly, ubiquitination has emerged as a cellular language to precisely communicate between and within cells. Monoubiquitination is the most basic form of ubiquitination and may occur on multiple Lys residues in the same protein to yield a multi-monoubiquitination. Owing to the type of linkage involved, ubiquitin chains can either be homotypic or heterotypic. In homotypic chains, all building blocks of the chain are linked through the same Lys or Met residues. However, heterotypic chains contain mixed and branched-type polyUb chains with different linkages in a single polymer. Thus, ubiquitin with its eight potential attachment sites, present all over the surface, enables the conjugates to adopt distinct but dynamic conformations. Moreover, ubiquitin is subject to additional PTMs like phosphorylation, acetylation, and ribosylation, which further expand the functional repertoire of Ub code. Extensive crosstalk among these PTMs directs ubiquitin signal regulation and/or diversification. Various ubiquitin modifications are recognized by effector proteins with linkage-specific ubiquitin-binding domains (UBDs), which couple the modified substrate with the downstream events.

Proteomic analysis of the Arabidopsis ubiquitylome demonstrated that six of seven homogeneous substrate-bound Lys chains were in an order of abundance of Lys_48_>Lys_63_>Lys_11_, followed by lower levels of Lys_33_>Lys_6_>Lys_29_ (Kim et al., 2013). Lys_48_ predominantly signals for proteasome-mediated degradation and is implicated in almost all aspects of plant signaling (Sadanandom et al., 2012; Walsh and Sadanandom, 2014). Lys_63_-linked ubiquitin chains can also generate proteasomal degrons (Saeki et al., 2009) but mostly targets modified proteins to lysosome and vacuole for degradation (Welchman et al., 2005; Kirkin et al., 2009). Plants display similar abundance and patterns of the abovementioned ubiquitin modifications responsible for many cellular processes, including endocytic sorting, DNA repair, plant immunity, and plant nutritional deficiency responses (Miricescu et al., 2018). Additionally, a Ub linkage type, Lys_29_, reportedly regulate the gibberellic acid (GA) pathway that signals for proteasomal degradation of DELLA proteins (Wang et al., 2009a). Other chains linked through Lys_6_, Lys_11_, and Lys_33_ are atypical to plants and have not been well-characterized. In addition to the polyubiquitin chain types, monoubiquitination is also responsive to various proteolytic (UPS or autophagy), as well as non-proteolytic signals, such as protein-protein interaction, localization, and activity modulation. Studies suggest that monoubiquitination of the histone H2B catalyzed by the E3 ligases, HISTONE MONOUBIQUITINYLATION 1 and 2 (HUB1 and HUB2), mediate plant immunity. Remarkably, to interpret and integrate the myriad of signals generated by the ubiquitin system, cells have evolved a range of specific UBDs that recognize multifaceted ubiquitin codes for different functions, primarily on the basis of a defined geometric assembly of different ubiquitin surfaces to trigger specific biological responses.

### The ubiquitin 26S proteasome system (UPS)

The UPS is a large (~2.5 MDa), multimeric ATP-dependent protease complex in all eukaryotic cells and serves as an elegant junk protein disposal system. It coordinates the abundance of various regulatory proteins involved in a myriad of signaling and metabolic pathways. Chaperon-assisted recognition and UPS-mediated degradation of aberrant proteins produced after translational errors or stresses emphasize the significance of UPS in protein quality control (Marshall and Vierstra, 2019). Since the proteasome is a negative regulator of the proteome, its regulation is critical for preserving homeostasis. Regulation of proteasome activity occurs at different levels, starting from its abundance and subcellular localization to proteolytic activity and post-function destruction, either through degradation of individual subunits or by removal of the proteasome as a whole (Livneh et al., 2016).

Substrate processing is a prerequisite for efficient protein degradation. The UPS combines strict substrate selectivity with extreme promiscuity for substrate processing to enable the degradation of thousands of proteins with high specificity. Importantly, tagging of proteins with Ubs is insufficient to label them for degradation. Apart from the protein sequence that determines ubiquitination, a loosely folded region near the end of the polypeptide is also required to determine the susceptibility of the target protein to proteasome for degradation. Thus, selective recognition of the Ub chain along with the poorly folded region is a fundamental basis for proteasomes to discriminate the target proteins for degradation from no-target proteins. Moreover, substrates' commitment to degradation is a critical and irreversible association between substrate and proteasome through its ATPase subunits, which, upon activation, drive the co-translational unfolding of substrates. The unstructured, unfolding-prone regions also serve as a starting point for translocation. Before the commitment, substrates undergo a reversible association with proteasome through intrinsic Ub receptors of 19S regulatory particle (RP) and facilitate their deubiquitination *via* DUBs. The majority of the substrates accomplish this binding to get deubiquitinated, but only those that undergo irreversible binding are designated as degron. Therefore, a kinetic competition between deubiquitination, release of the substrate, and allosteric activation of RP determine the fate of target proteins (Lee et al., 2001; Prakash et al., 2004; Peth et al., 2010; Yu et al., 2016; Collins and Goldberg, 2017).

### UPS-dependent regulation of abiotic stresses in plants

Plants utilize the activity of UPS to combat various abiotic stresses by regulating the turnover of short-lived post-functional regulatory proteins or stress-induced damaged proteins to mitigate the effects of dynamic environmental stresses. Transcriptome and proteome studies in different abiotic stress-exposed plants showed altered levels of TFs, modulating the expression profile of stress-responsive genes (Ellis et al., 2002; Dooki et al., 2006; Zhang and Xie, 2007; Zhu et al., 2007; Guo et al., 2008; Pandey et al., 2008; Sharma and Pandey, 2019). Over-expression of mono/polyubiquitin genes was reported to independently modulate stress tolerance in different organs of Arabidopsis (Sun and Callis, 1997), maize (Christensen et al., 1992), tobacco (Genschik et al., 1992; Lyzenga and Stone, 2012), and potato (Garbarino et al., 1992).

The considerable number and functional diversity of E3 ligases indicate their significance in the modulation of multiple abiotic stress-induced responses. Various enzymes involved in hormone biosynthesis, the abundance of related TFs, and other effector proteins are potential substrates of E3 ligases. The Arabidopsis DELLA proteins, known to suppress GA signaling, are the typical example of ubiquitination-dependent regulation of hormone effect as degradation of DELLA proteins in presence of GA involves SCF^SLY/GID2^ E3 ligase complex (Dill et al., 2004). The most common way employed by E3 ligases to regulate stress-related factors is to either act as a negative regulator that suppresses stress response pathways by targeting positive regulators for degradation or promotes stress signaling as positive response regulators that target negative regulators for degradation following stress perception or attenuate stress signaling by targeting positive regulators for degradation. In addition to E3, many E2 encoding genes are also stress-inducible. Transcript abundance of UBC2 in soybean (*GmUBC2*), groundnut (*AhUBC2*), and Arabidopsis (*AtUBC32*) was upregulated under water and/or salt stress (Zhou et al., 2010; Wan et al., 2011; Cui et al., 2012). Surprisingly, overexpression of *AtUBC32* rendered the plants sensitive to salt stress (Cui et al., 2012), while *atubc32* mutant plants were more tolerant to salt stress. Overexpression of mung bean UBC1 (*VrUBC1*), peanut (*AhUBC2*), or soybean (*GmUBC2*) genes in Arabidopsis plants showed improved tolerance to drought stress (Zhou et al., 2010; Wan et al., 2011; Chung et al., 2013). Overexpression of non-canonical cucumber Lys_63_-linked polyubiquitinating conjugase (CsUBC13) in Arabidopsis regulates the Fe-responsive gene(s) and promotes root development under iron-deficient conditions (Li and Schmidt, 2010). Transgenic tobacco overexpressing wheat monoubiquitin gene, *Ta-Ub2*, ameliorates the salt, cold, and drought stress (Kang et al., 2016), and improves photosynthesis under high light stress (Tian et al., 2014). Wheat F-box protein gene *TaFBA1*, a core component of the SCF E3 ligase complex, provides heat tolerance (Li et al., 2018).

Altered UPS activity can also affect the plants' tolerance to various environmental stresses. Mutations in UPS, especially, in the RP subunits, decrease complex accumulation, reduce the rate of ubiquitin-dependent proteolysis, and amend plant response to abiotic stresses (Smalle et al., 2003; Smalle and Vierstra, 2004; Ueda et al., 2004; Kurepa et al., 2008). Arabidopsis mutants of *rpn10-1, rpn1a-4*, and *rpn1a-5* were less tolerant to salt stress (Smalle et al., 2003; Wang et al., 2009b). Also, *rpn10-1* plants were hypersensitive to UV-radiation and DNA-damaging agents (Smalle et al., 2003). *Rpn1a-4, rpn1a-5, rpn10-1, rpn12a-1*, and *rpt2a-2* exhibited reduced heat-shock tolerance (Kurepa et al., 2008; Wang et al., 2009b). Hypersensitivity to various stress conditions shown by RP mutants suggests the crucial role of the proteasome in modulating plant responses to adverse growth conditions. Expression analysis of 67 *Triticum aestivum* α- and β-type subunits of 20S proteasome core protease at the seedling stage showed that 10 genes were involved in heat stress responses, 4 genes were involved in drought tolerance, and 9 genes were expressed under both heat and drought stress conditions, suggesting their active role in multiple abiotic stresses (Sharma et al., 2022). A significant reduction in the number of ubiquitinated proteins in soybean roots by flood stress suggests a relationship between proteasome-mediated proteolysis and waterlogging (Yanagawa and Komatsu, 2012). Direct or indirect disruption of UPS and its after-effects establishes its importance in plant responses to various stress conditions.

### E3 ligase function during drought stress

**Figure 4:**
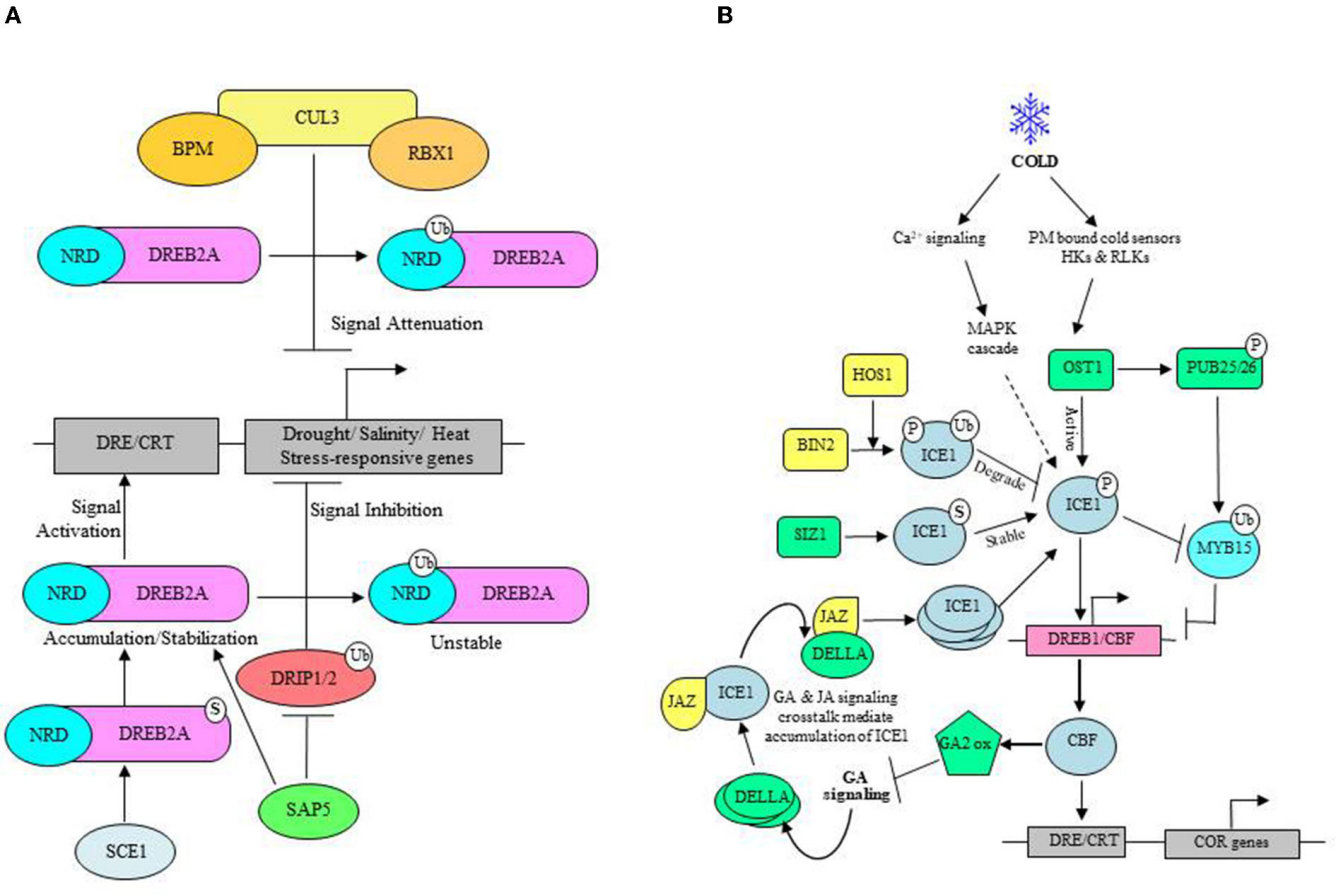
ABA-independent stress response. **(A)** Under drought and salt stress: DREB2A transcription factor functions upstream of various drought, salt, and heat-responsive genes. DRIP1/2 E3 ligase negatively regulates stress tolerance as a signaling inhibitor and targets DREB2A for UPS-mediated degradation. SAP5 E3 ligase reinforces stress signaling by regulating DRIP1/2 turnover. SCE1-mediated SUMOylation of DREB2A also enhances its stability. CUL3-based BPM ligases mark DREB2A for degradation as a signal attenuator. **(B)** Under cold stress: Cold stimulus is sensed by plasma membrane (PM) bound receptors, mostly histidine kinases (HKs) and receptor-like kinases (RLKs). Subsequent activation of Snf-related kinase (SnRKs), OST1, phosphorylates (P) ICE1, which enhances the expression of CBF and CBF-regulated cold stress-related (COR) genes. SIZ1 mediated SUMOylation (S) of ICE1 impedes the turnover rate and stabilizes ICE1. Cold stress-induced expression of MYB15 (a negative regulator of CBF) is ubiquitinated (Ub) and degraded *via* OST1 phosphorylated PUB25/26. BIN2 phosphorylated ICE1 levels are reduced by HOS1 E3 ligase in absence of stress. Cold stress-induced altered intracellular Ca^2+^ levels initiate MAPK signaling, which is also involved in ICE1 regulation, as shown by the dashed arrow. Crosstalk between GA and JA signaling also helps stabilize ICE1 wherein CBF stimulated GA2 oxidase inhibits bioactive GAs. This allows the accumulation of DELLA, which interacts and binds JAZ to release JAZ-bound ICE1, thereby promoting the accumulation of ICE1.

A well-described example of RING-type E3s is Dehydration Responsive Binding Element 2A (DREB2A) Interacting Protein1 (DRIP1) and DRIP2, which are involved in the regulation of stress-responsive ERF/AP2 transcription factor DREB2A that functions upstream of many drought- and salt-stress inducible genes (Figure 4A) (Qin et al., 2008). Accumulation of DREB2A only under drought conditions, *in vitro* ubiquitination of DREB2A by DRIP1, and increased stabilization of DREB2A in *drip1/drip2* double mutants indicate that the abundance of DREB2A is negatively regulated by DRIP1/2 (Sakuma et al., 2006a,b; Qin et al., 2008; Al-Saharin et al., 2022). Moreover, the increased abundance of DREB2A upon UPS inhibition and enhanced drought tolerance in *drip1/drip2* double mutants was concomitant with a significant increase in expression of several drought-inducible genes specifically regulated by DREB2A (Qin et al., 2008). It showed that DREB2A is unstable under non-stress conditions, and RING ligases DRIP1/2 function redundantly to suppress drought signaling *via* ubiquitin-mediated proteolysis of DREB2A. The presence of 30 residues' long serine and threonine-rich negative regulatory domain, the degron, makes DREB2A unstable and acts as a signal for ubiquitin-mediated degradation. In absence of stress, DRIP1/2 localizes in the nucleus, interacts with DREB2A, and directs the destruction of DREB2A (Qin et al., 2008). Under stress conditions, perhaps the DREB2A degron is made unavailable to degradation machinery, which leads to accumulation and stabilization of DREB2A. Since stress conditions do not affect the transcript levels of DRIP1/2, there is a possibility that drought-induced re-localization of DRIP1/2 to cytosol may lead to DREB2A stabilization. Stress Associated Protein5 (SAP5) is another E3 that functions upstream of DRIP1/2 ligases and promotes their degradation, thereby modulating drought-stress responses positively in Arabidopsis and wheat. Moreover, Arabidopsis SAP5 has been reported as a positive regulator of salt and osmotic stress tolerance (Al-Saharin et al., 2022; Table 3).

**Table 3:** List of abiotic stresses responsive E3 Ub ligases in plants.

**Stress type**	**RING**			**U box**	**CRLs**
Drought	AtRGLG1/5^a^ At/OsSAP5 AtAIRP1/2 AtAIRP3^a^ AtNERF AtRDUF1/2 AtSDIR1^a^ AtDUF1/2^a^ AtATL61/78^a^ AtJUL1	AtPPRT3^a^ AtRHA2B/2A^a^ AtRZP34/CHYR1 AtXBAT35.2^a^ AtXERICO^a^ AtRma1/2/3 OsRF1^a^ OsRDCPS OsRHP1 OsSDIR1	OsZNF CaASRF1 CaAIRE1 CaDTR1 CaRma1H1 CaATIR1^a^ CaAIRF1 ZmAIRP4 ZmRFP1	AtPUB12/13 AtPUB46/48 OsPUB67 TaPUB1 GmPUB6/8^a^	TaFBA1 AtDRS1 AtWDR55
	AtRGLG2^a^ AtMIEL^a^ AtRZF1 OsDSG1 OsDIS1 OsSADR1 OsDIRP1	OsDHSRP1 TaDIS1 CaAIR1^a^ CaREL1^a^ CaDIR1^a^ GmRFP1		AtPUB11 AtPUB18/19^a^ AtPUB22/23^a^ OsPUB41 CaPUB1 GmPUB21	AtDOR^a^ AtAFA1^a^ AtSDR AtPP2-B1 AtHOS15 AtRAE1^a^ OsCBE1
Salinity	AtSAP5 AtAIRP1/2^a^ AtAIRP3^a^ AtRDUF1/2 AtSDIR1^a^ AtSTRF1	OsRHP1 OsSIRF1 OsSIRP2 OsSIRH2-14 OsRMT1 OsRF1^a^	TaZNF ZmRFP1 MfSTMIR SpRING GmRFP1	AtPUB10 AtPUB15 TaPUB1 TaPUB15	AtSDR AtPP2-B11 TaFBA1
	AtXBAT35.2 AtPPRT1 OsDSG1 OsMAR1	OsSIRP1/3/4 OsSRFP1 OsSADR1 OsDIRP1	OsDHSRP1 GhSARP1 MdMIEL	AtPUB10^a^ AtPUB30 TaPUB26 GmPUB21	OsCBE1^a^
Cold	OsDIRP1 OsCOIN			OsPUB2/3 CaPUB1	-
	AtHOS1 AtATL61 AtATL78	AtATL80 OsSRFP1 OsHOS1	OsSRF GmRFP1	–	OsCBE1
Heat	AtPPRT1 OsHIRP1			AtPUB48	TaFBA1
	OsDHSRP1				
Oxidative	–			AtPUB46 OsPUB15	TaFBA1
	OsSRFP1 OsSRF MdMIEL			AtPQT3	–
Heavy metals	OsHIR1 OsAIR4.1/4.2 SlRING1 AtATRF1			TaPUB1	–
	OsHRZ				

Positive regulators and negative regulators have been shown with unshaded and shaded backgrounds, respectively.

“^*a*^” denotes ABA-dependence.

Plant U-box type E3s are also known to participate in the regulation of drought stress tolerance. For example, PUB11, by targeting Leucine-rich Repeat Protein1 (LRR1) and Kinase7 (KIN7), impedes stomatal closure and reduces drought tolerance. Other members of the U-box family that are known to downregulate drought tolerance are PUB19, PUB22, and PUB23 (Table 3). On contrary, PUB46 and PUB48 are reported as positive regulators of drought stress since their mutations lead to drought hypersensitivity, though their potential targets remain elusive (Al-Saharin et al., 2022). Rice PUB41 (OsPUB41) is known to have a negative influence on drought-responsive signaling as it mediates the degradation of Chloride Channel6 (OsCLC6), which is critical for modulating chloride homeostasis under water deficit conditions.

In *Capsicum annum*, the RING membrane-anchor 1 homolog 1 (Rma1H1) was originally identified as a dehydration-regulated gene (Park et al., 2003), which, when overexpressed in Arabidopsis, resulted in increased drought tolerance (Lee et al., 2009). The plasma membrane aquaporin PIP2/1 is known to serve as a potential target for Rma1H1 and was shown to interfere with a UPS inhibitor, MG132, suggesting the role of Rma1H1 in proteasome-mediated stress tolerance by regulating the aquaporin levels in plants. It has been suggested that aquaporins can negatively impact plants during water stress because they facilitate symplastic water transport (Jang et al., 2004; Alexandersson et al., 2005). Rma1-3 are the Arabidopsis homologs of Rma1H1 (Lee et al., 2009). Together, Rma1H1 and Rma1 promote drought tolerance by mediating the degradation of aquaporins. This contradicts CaPUB1 and its Arabidopsis homolog AtPUB22/23 since their over-expression renders the transgenic plants more sensitive to drought and salt conditions. The RP subunits, which are Rpn6 and Rpn12a, are the potential substrate for CaPUB1 and PUB22/23, respectively. The significance of these interactions is still unknown, but ubiquitin-dependent regulation of Rpn subunits may regulate the activity of the UPS during the drought stress response.

### Ubiquitination and salinity stress response

E3 ligases are also well-explored for their function in the regulation of salt-induced adaptive responses. Myriads of E3 ligases are reported that confer tolerance to more than one stress, such as drought and salt stresses, by regulating common factors functioning under both stresses. One shared factor is ABA, which is mainly a stress-induced hormone, that signals reprograming of cellular osmotic and ionic steady states to mitigate common physiological responses, such as stomatal movement regulation. In wheat, a U-box protein TaPUB15 has been implicated in positive salt stress tolerance (Table 3) by stabilizing the expression of stress-tolerant genes and maintaining low Na^+^/K^+^ ratios (Li et al., 2021). Conversely, PUB26 affects salt stress response negatively (Table 3) by interacting with one of the ATPase subunits of 26S proteasome, *T. aestivum* Regulatory Particle Triple-ATPase2A (TaRPT2a). Its gene expression is salt inducible, but the molecular mechanism underlying this interaction requires further studies (Al-Saharin et al., 2022). SALT TOLERANCE RING FINGER 1 (STRF1) is a membrane-associated RING-H2 type E3 ligase annotated as a positive modulator of salt stress in Arabidopsis (Table 3). It localizes to intracellular endosomes and confers salt stress by regulating membrane trafficking and ROS production (Tian et al., 2015). In rice, four members of the SALT-INDUCED RING PROTEIN (SIRP) family of RING-type E3 ligase have been identified. Overexpression of SIRP1/3/4 increased the sensitivity of transgenic plants toward salt stress, which indicated their roles as negative modulators of salt stress tolerance, whereas SIRP2 overexpressed plants with enhanced salt resistance suggested a positive role for SIRP2. While substrates of SIRP1 are not known, SIRP3 and SIRP4 are known to impose their inhibitory effects by facilitating the degradation of stress-induced positive regulators. While OsSIRP3 targets MADS-BOX GENE 70 (OsMADS70) and an ABC DOMAIN CONTAINING PROTEIN (OsABC1P11), SIRP4 triggers proteasomal degradation of PEROXISOMAL BIOGENESIS FACTOR11-1 (OsPEX11-1). The *O. sativa* Transketolase 1 (OsTKL1) has been identified as a potential target of OsSIRP2; however, the significance of ubiquitination-mediated degradation of OsTKL1 under salt stress is unclear (Al-Saharin et al., 2022).

### Ubiquitination and temperature stress response

UPS also functions to attenuate cold stress signaling as exemplified by High expression of osmotically responsive gene1 (HOS1) RING-type E3 engaged in degradation of ICE1 (Inducer of CBF expression 1), a MYC transcription factor that controls the expression of cold-responsive transcription factor DREB1A/C-REPEAT BINDING FACTOR3 (CBF3) involved in the regulation of numerous cold-responsive genes (Figure 4B). Consistent with the role in mediating ICE1 protein turnover, overexpression of HOS1 suppresses the expression of ICE target genes while increasing the sensitivity to freezing conditions. Although HOS1 possesses a variant RING domain, it catalyzes *in vitro* and *in vivo* ubiquitination of cold-responsive ICE1 (Lee et al., 2001; Stone et al., 2005; Dong et al., 2006). Interestingly, both the upregulation and downregulation of ICE1 TF is a cold-induced phenomenon. However, cold-inducible genes are only transiently expressed (Chinnusamy et al., 2003) and facilitated by cold-induced relocalization of HOS1 from the cytoplasm to the nucleus, enabling proteasomal degradation of nucleus-localized ICE1 (Lee et al., 2001; Dong et al., 2006). Critical regulation of ICE1 activity by phosphorylation indirectly modulates its stability through UPS. High temperature also engages E3s to monitor the abundance and the activity of several TFs that promote or suppress transcription of stress-related genes to activate appropriate mitigation responses. Heat stimulus-based physiological responses involve increased production of ROS, alteration in protein structure, and perturbed membrane integrity due to lipid peroxidation. Mitigation of heat stress essentially requires transcriptional activation of thermotolerance-related genes and stabilization of upstream transcriptional activators. For example, A RING-finger E3 ligase in Arabidopsis known as PROTEIN WITH RING DOMAIN AND TMEMB1 (AtPPRT1) induces the expression of the following heat-responsive genes: HEAT SHOCK PROTEIN 21 (AtHSP21), HEAT SHOCK TRANSCRIPTION FACTOR A7A (AtHSFA7a), and ZINC-FINGER PROTEIN 12 (AtZAT12) by targeting transcriptional repressors of these genes. Related studies elucidated the role of AtPPRT1 in the negative regulation of drought and salt stress tolerance (Table 3). Similarly, overexpression of AtPUB48, a U-box type E3, acts as a positive regulator of heat stress-responsive genes and increases their expression. Following the fundamental theme of imparting stress tolerance by promoting turnover of negative elements and stabilization of positive elements, rice RING E3 ligase, HEAT-INDUCED RING FINGER PROTEIN1 (OsHIRP1), show heat stress tolerance through proteasomal degradation of ALDO/KETO REDUCTASE4 (OsARK4) and HIRP1-REGULATED KINASE1 (OsHRK1) that likely affects heat-stress tolerance negatively (Al-Saharin et al., 2022).

### Ubiquitination and UV radiation stress response

A significant amount of UV-B (280–320 nm) reaching the earth's surface is another source of abiotic stress. High levels of UV-B exposure generate reactive oxygen species (ROS) that lead to DNA damage. Conversely, low levels of UV-B act as a signal that triggers regulatory responses involved in repairing UV damage. Two basic mechanisms of DNA-damage repair are photoreactivation and nucleotide excision repair (NER) (Tuteja et al., 2009). The NER pathway involves a CUL4-DNA Damage Binding protein1 (DDB1)-based CRL that targets a nucleus-localized DDB2 protein, which binds to UV-induced bulky DNA lesions (Molinier et al., 2008). Nuclear translocation of DDB1, following UV irradiation, facilitates the degradation of DDB2. CUL4-DDB1-mediated removal of DDB2 from DNA lesions recruits and permits NER machinery to access the lesions. Ataxia Telangiectasia-mutated and Rad3-related (ATR) protein kinase, as well as De-etiolated1 (DET1) factors, assist in UV-induced CUL4-DDB1-mediated degradation of DDB2. ATR identifies the damaged DNA and ensures the presence of DDB1 in the nucleus through DET1 for the degradation of DDB2. DET1 degradation also occurs along with DDB2 in a CUL-DDB1-dependent manner. Overexpression of DDB1A and DDB2 in Arabidopsis was reported to enhance the UV-C tolerance (Molinier et al., 2008).

### Ubiquitination and nutrient deprivation stress response

The role of UPS in mitigating stress-stimulated adverse effects extends beyond the proteolysis of TFs. Availability of nutrients after germination is crucial in determining whether the seedling can transit through the post-germinative developmental checkpoint or not (Lopez-Molina et al., 2001). The RING-type E3, Arabidopsis Toxicos EN Levadura6 (ATL6 and ATL31), are involved in the regulation of 14-3-3 proteins. The 14-3-3, are conserved regulatory proteins in eukaryotes involved in a multitude of signaling processes. Overexpression of 14-3-3χ protein, a target of ATL31 ubiquitin ligase, results in hypersensitivity to C/N stress (Sato et al., 2011; Maekawa et al., 2012). On this account, loss of ATL6 and ATL31 produce hypersensitivity to C/N stress, while over-expression of ATL6 and ATL31 rendered plants insensitive to C/N stress such that these transgenic plants were able to bypass early checkpoints despite the stress conditions (Sato et al., 2009). Importantly, C/N stress-induced accumulation of 14-3-3χ is observed in wild-type seedlings but not in *atl6atl31* seedlings, suggesting that ATL6/31 mediates the turnover of 14-3-3χ under non-stress conditions and degradation is prohibited during exposure to C/N stress.

Plants adapt to nitrogen-limiting conditions by redistributing nitrogen content from older to younger, actively growing organs and increasing the accumulation of anthocyanins (Kant et al., 2011). The adaptive response of plants to low nitrogen is facilitated by the presence of Nitrogen Limitation Adaptation (NLA), a RING-type ligase (Peng et al., 2007).

### Ubiquitination in ABA-mediated stress response

Drought, cold, and salinity stress-activated signal transduction pathways share several related components that are linked with the ABA-mediated stress response and are regulated in a UPS-dependent manner. ABA is an important plant hormone that controls vital cellular and physiological responses in development and abiotic stresses. As a negative regulator of growth and development, ABA promotes dormancy, regulates seed maturation to ensure germination only under growth-promoting conditions, and suspends growth in seedlings exposed to stresses. ABA also mediates protective responses that mitigate the stress-induced damage in mature plants (Finkelstein et al., 2002; Himmelbach et al., 2003). ABA-dependent regulation of stomatal closure in response to water deficiency is a typical example of protective responses of ABA in plants to prevent excessive water loss.

**Figure 5:**
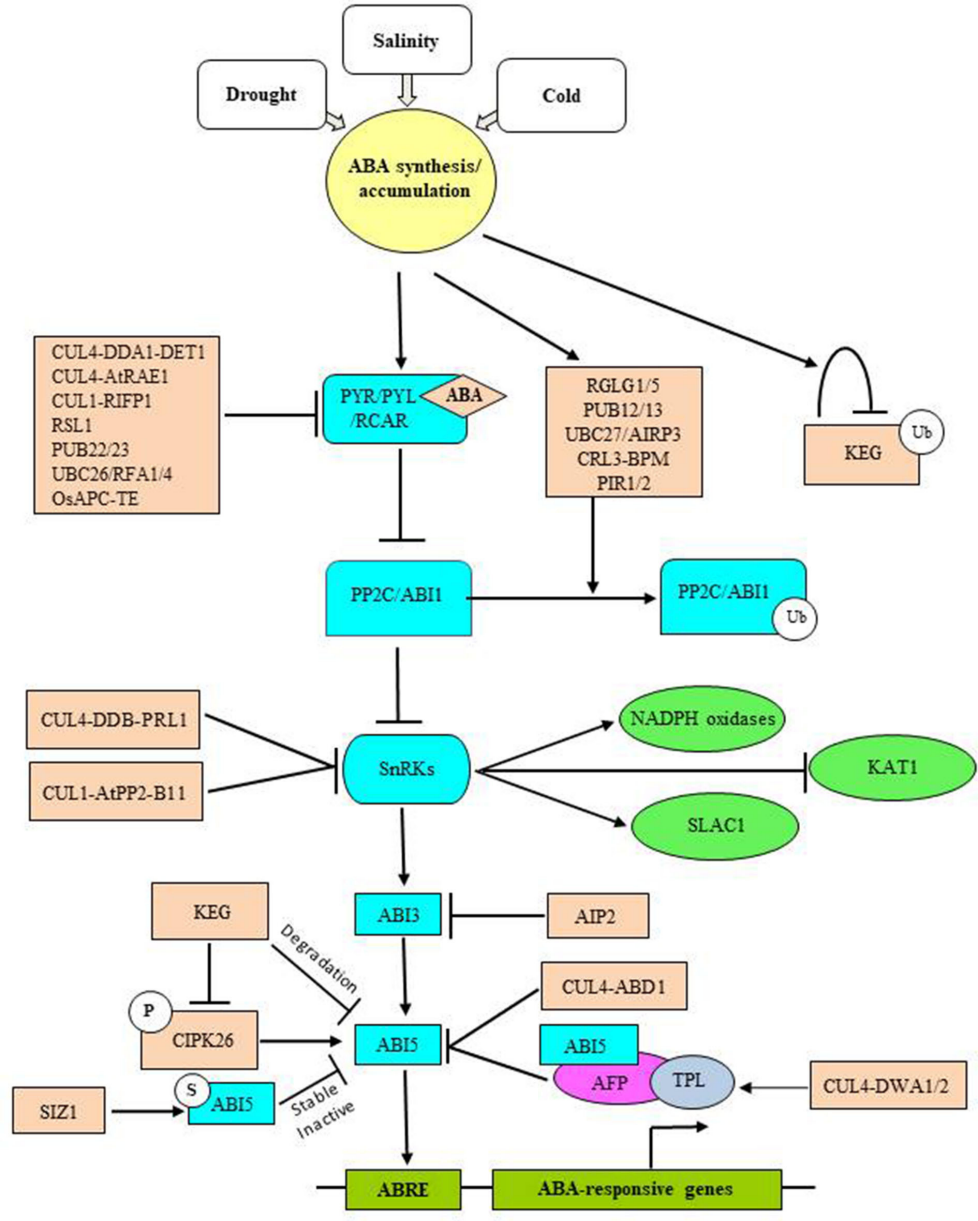
UPS-mediated ABA-dependent stress signaling/tolerance in plants. Abiotic stress triggers ABA biosynthesis and accumulation. In the absence of stress conditions, the positive regulators of ABA signaling like ABA receptors and TFs are maintained in a repressed state to inhibit ABA signaling and thus the expression of ABA-responsive genes. This is largely achieved through numerous E3 ligases that differentially modulate the turnover of ABA receptors and SnRKs, as these are major effectors of ABA responses. SIZ1-mediated SUMOylation opposes KEG-mediated degradation of ABI5 to maintain moderate ABI5 levels under no stress conditions. Auto-ubiquitination and degradation of KEG under-enhanced ABA levels stabilize ABI5. Upon ABA accumulation, ABA binds its receptors, which together capture PP2C in a ternary complex to release PP2C imposed inhibition of SnRKs. PP2Cs also serve as targets for E3 ligases. Fully activated SnRKs then induce ABI3 activity, which turns on ABI5 functions. Other TFs like ABFs are also activated in the same manner. SnRKs also activate ROS scavenging enzymes (NADPH oxidases) and anion efflux channels (SLAC1), as well as inhibit K^+^ influx channels (KAT1), to mitigate ROS and osmotic effects. Attenuation of ABA signaling requires UPS-mediated turnover of ABA TFs. As mentioned above, ABI3 functions upstream of ABI5 and is degraded by AIP2, whereas degradation of ABI5 employs CUL4-based ABD1 and DWA1/2 E3 ligases. DWA1/2-mediated breakdown of ABI5 occurs *via* ABI5-binding protein (AFP), which recruits the co-repressor of JA signaling, TOPLESS (TPL) to ABI5, to generate a transcriptional complex that represses the expression of ABA-responsive genes.

Stresses stimulate ABA biosynthesis, which signals the expression of hundreds of ABA-responsive genes. ABA-responsive TFs, like basic leucine zipper (bZIP), alter gene expression by interacting with ABA-regulatory elements (ABRE) in the promoter region of stress-responsive genes (Hattori et al., 2002; Narusaka et al., 2003). UPS-mediated regulation of ABA signaling is achieved by modulating the stability of ABRE-binding TFs (Figure 5). ABA promotes the accumulation of short-lived bZIP TF Abscisic Acid Insensitive 5 (ABI5), which serves as an early developmental checkpoint for the growth of young seedlings under adverse conditions (Uno et al., 2000; Lopez-Molina et al., 2003). The abundance of ABI5 is regulated by Keep on Going (KEG), a trans-Golgi network/cytosol localized RING E3, which directs ubiquitination and degradation of ABI5 (Stone et al., 2007). Under non-stress conditions, KEG maintains low levels of ABI5 in the cytosol, which prevents the accumulation of ABI5 in the nucleus and attenuates ABA signaling to ensure seedling establishment. Conversely, elevated levels of ABA accelerate self-ubiquitination and degradation of KEG, which leads to the ABI5 stabilization and promotion of ABA responses. This suggests a feedback loop-like mechanism for ABI5 and KEG-mediated ABA signaling. Other bZIP TFs, like ABRE-binding factor 1 (ABF1) and ABF3, also serve as potential substrates for KEG.

E3 ligases, DWD-hypersensitive to ABA 1 (DWA1) and DWA2, have also been implicated in the regulation of ABI5 turnover. These are substrate-recruiting components of CUL4-based CRL and function as negative regulators of ABA signaling by targeting ABI5 for UPS-mediated degradation. Interestingly, ABI5 accumulation is observed only in ABA-treated *dwa1/2* mutants, which contradicts with *keg* mutants that show extremely high levels of ABI5 even in the absence of ABA. Thus, it suggests that KEG may function to maintain low levels of ABI5 in the absence of ABA and abiotic stress, while DWA1/2 may function to attenuate ABA signaling so that plants can readily re-establish growth once environmental conditions improve. DWA1/2-mediated breakdown of ABI5 has been proposed to occur *via* ABI5 binding protein (AFP), which adds further complexity to the UPS-mediated regulation of ABI5.

UPS-regulated ABI3, an ABA-responsive B3 type TF, functions upstream of ABI5 to mediate ABA-dependent responses (Finkelstein and Lynch, 2000; Lopez-Molina et al., 2002). RING-type E3 ABI3-interacting protein (AIP2) is a negative regulator of ABA signaling and targets ABI3 for proteasomal degradation to inhibit the ABA responses. The ABA-induced abundance of AIP2 suggests that ABA promotes the turnover of ABI3 *via* AIP2. Studies also suggested a proteolytic regulation for ABI4, ABF2, and ABF3 TFs. ABA-dependent stabilization of ABF3 involves phosphorylation of ABF3 *via* SnRK2 kinase, the Open Stomata 1 (OST1). Stabilization of ABF3 in response to phosphorylation demonstrates that ABA-activated kinases are not limited to the activation of TFs, but they may also be required for stability.

Moreover, RING-type E3 ligases, such as SDIR1, AtAIRP1, ATL61, RHA2a, and RHA2b, are shown to be the positive regulators (Table 3) of ABA signaling (Al-Saharin et al., 2022). Transgenic lines over-expressing these E3 ligases are hypersensitive to ABA-induced effects and were more tolerant to drought, while mutant plants were insensitive (Zhang et al., 2007; Bu et al., 2009; Ryu et al., 2010; Li et al., 2011; Yang et al., 2020). Overexpression of SDIR1, AtAIRP1, or RHA2b enhances drought tolerance *via* an increase in ABA-induced stomatal closure (Zhang et al., 2007; Ryu et al., 2010; Li et al., 2011). Expression of SDIR1 is induced in response to salt and drought conditions and functions upstream of ABA-responsive TFs ABI3 and ABI5 by degrading the SDIR INTERACTING PROTEIN 1 (SDIRIP1) (Shu and Yang, 2017). Unlike the aforementioned ligases, RHA2a and RHA2b are redundant in nature and function parallel to ABA-responsive TFs ABI3 and ABI5 (Bu et al., 2009; Li et al., 2011). Drought Tolerance Repressor 1 (DOR1), an F-box component of SCF E3 ligase complex in Arabidopsis, is reported as a negative regulator of ABA-mediated stomatal closure since *dor1* is hypersensitive to ABA with enhanced stomatal closure, hence improving stress tolerance. Nine *cis*-epoxycarotenoid dioxygenase 3 (NCED3*)*, a key enzyme in ABA biosynthesis, is significantly upregulated in *dor* plants (Zhang et al., 2008). Another RING-type E3 linked to ABA biosynthesis is XERICO, whose overexpression produced ABA hypersensitive drought-resistant plants. A stronger and more sustained expression of NCED3 in XERICO, over-expressing plants upon ABA treatment, indicates that XERICO acts post-translationally to regulate ABA biosynthesis (Ko et al., 2006). Recently, novel RING-H2 types E3 in rice OsRF1, a homolog of Arabidopsis AtXerico, have been reported to elicit ABA biosynthesis and accumulation against drought. OsRF1 is also a positive player in salinity stress. A member of clade A PP2C protein family, OsPP2C09, which is a core suppressor of ABA signaling, has been identified as a potential target of OsRF1. Thus, OsRF1 confers stress tolerance in an ABA-dependent manner (Kim et al., 2022). Arabidopsis Ring Domain Ligase1 (RGLG1) and its homologs, RGLG2 and RGLG5, synchronize ABA-mediated plant responses during drought stress due to their dual but opposing behavior (Wu et al., 2016). RGLG2 signals UPS-mediated degradation of the positive regulators of drought signaling, Ethylene Response Factor53 (AtERF53) and MAPKKK18, to downregulate stress responses, whereas RGLG1 targets the repressor of drought-induced ABA signaling, PP2CA, to promote ABA-dependent drought tolerance. Upregulation of ABA signaling *via* RGLG1 involves ABA-mediated nuclear localization of RGLG1 by inhibiting N-myristoyltransferase 1 (NMT1), which, otherwise, myristoylate and target RGLG1 to the plasma membrane (Al-Saharin et al., 2022).

### Ubiquitination in submergence stress response

Submergence gives rise to multiple stress conditions that occur simultaneously like hypoxia, decreased gaseous exchange, altered osmoticum, low transpiration, nutrient deficiency, oxidative, and salt stress (Tamang and Fukao, 2015; Fukao et al., 2019). Similarly, post-submergence plants are suddenly exposed to normoxia, photoinhibition, oxidative stress, and dehydration (Tamang and Fukao, 2015). Plants respond to transient deep flash floods through quiescence response while following escape response by rapid internodal elongation under slow progressive floods. Quiescence response involves economization of energy sources by suppressing GA signaling in brassinosteroids (BR)-dependent manner. This also involves the *SUBMERGENCE1A* (*SUB1A*) which is a member of ETHYLENE RESPONSIVE FACTOR (ERF- VII) family. The ethylene-dependent abundance of SUB1A promotes BR biosynthesis, which leads to the degradation of bioactive GAs and subsequent accumulation of DELLAs. However, *SNORKEL* (*SKs*), the antagonist of *SUB1A* although accumulates in an ethylene-dependent manner, downregulates BR production, which reverses the action of BR resulting in enhanced GA signaling and acquisition of escape response. There are five *ERF-VII* genes in Arabidopsis, two *HYPOXIA RESPONSIVE ERF* (*HRE*) genes, *HRE1* (*ERF73*) and *HRE2* (*ERF71*), and three *RELATED TO AP2* (*RAP2*) genes, *RAP2*.2 (*ERF75*), *RAP2*.3 (*ERF72*/*EBP*), and *RAP2*.*12* (*ERF74*), and all have been shown to promote submergence tolerance and oxygen deprivation. The UPS is implicated in the modulation of ERF-VII protein abundance *via* the N-end rule pathway. In this pathway, the constitutive cleavage of methionine from ERF-VII proteins conserved N-terminus, Met-Cys, by methionine aminopeptidases exposes cysteine, which in the presence of oxygen and NO, is oxidized by plant cysteine oxidases (PCOs), thereby producing cysteine sulfinic or cysteine sulfonic acid. Subsequent conjugation of oxidized cysteine with an arginine residue of arginyl tRNA transferases (ATEs) serves as a recognition and ubiquitination site for E3 Ub ligase, PROTEOLYSIS6 (PRT6), which signals proteasome-mediated degradation of ERF-VII proteins. While the *SUB1A* gene is associated with submergence tolerance in rice (Fukao et al., 2011), another APETELLA2/ETHYLENE RESPONSIVE FACTOR (AP2/ERF) type TF EREBP1 confers adaption to submergence, as well as drought in rice (Jisha et al., 2015). Another E3 Ub ligase SUMERGENCE RESISTANCE1 (*SR1*) in Arabidopsis has been identified as a modulator of submergence tolerance, wherein it synchronizes plant growth with stress acclimation through a switch module, including SR1-WRKY33-RAP2.2. The stress-induced phosphorylation of WRKY33 facilitates downstream activation of RAP2.2, which, in turn, induces the expression of hypoxia-related genes. Upon reoxygenation, SR1 catalyzes the ubiquitination and degradation of WRKY33, while RAP2.2 is degraded through the N-end rule pathway (Liu et al., 2021). Similarly, phosphorylation and ubiquitination negatively regulate the function and stability of nitrate reductase (NR) in normoxia (Kim et al., 2018; Fukao et al., 2019). The ubiquitin ligase, CONSTITUTIVE PHOTOMORPHOGENIC 1 (COP1), reportedly destabilizes the NR structure (Park et al., 2011). However, SUMOylation positively affects the stability of NR protein in normoxia by partitioning its subcellular location to the nucleus (Park et al., 2011; Kim et al., 2018). In the comparison of submergence-sensitive *Rumex acetosa* with submergence-tolerant *Rhodopseudomonas palustris*, plants of tolerant species showed higher expression of COP1 and nonsymbiotic hemoglobin concomitant with lower ammonia, higher nitrate, and protein concentrations even in normoxia due to post-translational suppression of NR through ubiquitination (van Veen et al., 2013).

### Ubiquitination in epigenetic regulation of abiotic stresses

Although the intricate relationship between the chromatin remodeling dynamics and ubiquitin code is arduous, the epigenetic regulatory networks of histone modification play a vital role in genome stability, thereby regulating abiotic stress responses in plants. Lysine-rich histones are often ubiquitinated and play a critical role in DNA damage responses triggered in response to double-strand breaks (DSBs) due to genotoxic stresses (Mattiroli and Penengo, 2021). The H2A and H2B are the most abundant monoubiquitinated histone types and evoke an immediate response to DNA damages. Recruitment of Ub ligase RNF-8 and RNF168 at sites neighboring DSBs catalyzes K-63, K-27, and monoubiquitination of K13/15 in H2A type histones. Ubiquitinated histones then serve as docking sites for downstream repair machinery. RNF168-mediated ubiquitination of H2A is signaled by ataxia-telangiectasia mutated (ATM)-mediated phosphorylation of H2AX (H2A variant), followed by recruitment of RNF8, which catalyzes ubiquitination of H1 linker histones (Aquila and Atanassov, 2020). Studies report the implication of H2B monoubiquitination in abiotic stress tolerance in Arabidopsis and rice. For H2B ubiquitination, the Arabidopsis genome encodes two RING E3 ligases, HISTONE MONOUBIQUITINATION1 (HUB1) and HUB2, and three E2 conjugases, UBIQUITIN CARRIER PROTEIN1 (UBC1), UBC2, and UBC3 (Cao et al., 2008). The *hub1* and *hub2* mutants showed decreased H2B ubiquitination and increased sensitivity to salt stress. It demonstrates that H2Bub is involved in salt stress-induced microtubule depolymerization. Further, mutants showed downregulation of genes encoding for cutin and wax biosynthesis, revealing their potential function as positive regulators of drought tolerance. Consistently, the ectopic expression of AtHUB2 in transgenic cotton conferred improved tolerance to drought stress. Similarly, OsHUB2 overexpression enhanced the ABA sensitivity and drought resistance in rice, suggesting that OsHUB2 monoubiquitination positively modulates drought tolerance in an ABA-dependent manner (Ueda and Seki, 2020).

## SUMOylation

Eukaryotic cells employ a variety of small polypeptides as post-translational regulators of protein function. SUMOylation is another crucial PTM in eukaryotes that redefines the fate of various proteins by altering their intra- and inter-molecular interactions. Small Ub-related protein Modifiers (SUMOs) are small polypeptides of 10 kDa, which, like ubiquitin, attach to accessible lysine(s) of target proteins through an isopeptide bond. Lysine within ψKXE/D (ψ denotes bulky hydrophobic group and X represents any amino acid) motif is the minimal consensus required for the attachment of SUMO to a lysine residue in the substrate. Nascent SUMO polypeptides have a variable C-terminal extension cleaved by SUMO-specific proteases, a prerequisite for conjugation, to generate mature SUMO proteins with a signature glycine di-peptide at C-terminus implied in isopeptide bond formation. Although SUMO peptide folds into the ubiquitin-like β-grasp fold, they share only 20% sequence identity with ubiquitin and exhibit a completely different surface charge distribution. This might be because SUMOs evolved to have functional specialization while conserving the fundamentals of covalent PTMs. SUMO is unique to have 10–30 residue N-terminal extensions associated with polySUMOylation (Geiss-Friedlander and Melchior, 2007; Srivastava and Sadanandom, 2020). This intrinsically disordered structure seems to provide enough flexibility and solvent exposure, facilitating its modification *via* other PTMs or its attachment to host proteins. Apart from canonical SUMOs, plants express certain atypical SUMO forms that are tissue and/or condition-specific or lack signature Gly-Gly motif at C-terminus with an unusually long N-terminus (SUMO-variant/SUMO-v). Another variant with two β-grasp folds in tandem, known as diSUMO-like (DSUL), is exclusive to cereals. The highly conserved paralogs and DSULs have been identified as the only conjugating forms, the former being the widely expressed forms directing most of the functions (Augustine and Vierstra, 2018). There are a few other ubiquitin-like proteins, such as Ubiquitin fold modifier (UFM), Related to ubiquitin 1(RUB1), and homology to ubiquitin (HUB), that primarily regulate the protein activity, subcellular localization, and protein-protein interactions. The ubiquitous presence and conservation of SUMOylation across the eukaryotes signify its crucial role in sustaining proteome functions. Yeast, *C. elegans*, and *D. melanogaster* contain only one SUMO coding gene, while higher organisms have expanded gene families with several genes. For example, the human genome has four SUMO genes while Arabidopsis has eight isoforms, of which only the expressions of SUMO1, 2, 3, and 5 have been verified (Srivastava and Sadanandom, 2020). Non-complementation of *sumo1/sumo2* double mutants by SUMO3/5 suggests its non-redundant functions. However, SUMO1 and SUMO2 with 83% identity are known to complement each other. Less conserved SUMO3 and 5 share only 42 and 30% similarity with SUMO1, respectively. Constitutive expression studies of SUMO3/5, showing delayed growth and senescence-like symptoms, suggest a strict control over their expression for optimal growth in plants. No such growth defects were observed for SUMO1/2 constitutive expression. The rice genome has a repertoire of six SUMO genes (*SUMO1-6*). All isoforms were shown to rescue SUMO-deficient UV-sensitive *pmt3*Δ mutants of fission yeast. Of these, *SUMO3-6* contains SUMO-acceptor site ψKXE/D, indicating their involvement in the formation of SUMO chains (Teramura et al., 2021). *AtSUMO1/2* and *OsSUMO1/2* are plant homologs of human *SUMO2/3* (Miura et al., 2007).

Arabidopsis proteome has thousands of SUMO-target proteins suggesting SUMOylation as a major PTM like phosphorylation and ubiquitination (Augustine and Vierstra, 2018). Enhanced SUMOylation of nuclear proteins is among the most rapid response to various stresses but the precise mechanism of stress resilience through this PTM remains ambiguous. A major portion of identified SUMO substrates is nuclear proteins, such as TFs, coactivator/repressors, and chromatin modifiers, which indicate the imperative role of SUMOylation in regulating nuclear processes. For instance, SUMOylation controls nucleo-cytoplasmic trafficking of RanGAP1, its unmodified form is cytosolic, whereas modified RanGAP1 interacts with nucleoporin NUP358 (RanBP2) for its nuclear localization (Geiss-Friedlander and Melchior, 2007). The reversibility of SUMOylation is crucial for its regulatory role and is mediated by deSUMOylating proteases (DSPs). The higher number of DSPs than E3s in plants suggests that selectivity is important for SUMOylation but is critical to deSUMOylation as latter is required for the maintenance of a dynamic pool of SUMOylated and non-SUMOylated proteins to manifest the diverse responses (Augustine and Vierstra, 2018). The eukaryotic proteome is ~15–20% SUMOylated. Three major functions of SUMOylome include regulating activity and localization of individual proteins; macromolecule biosynthesis; and SUMO-mediated proteasomal degradation (Drabikowski et al., 2018; Morrell and Sadanandom, 2019). SUMOylation-directed degradation of proteins involves SUMO-targeted Ub-ligases (STUBLs) comprising a novel class of Ub ligases that recognize and ubiquitinates SUMO-tagged proteins for proteasome degradation. The STUBLs essentially interact non-covalently with polySUMOylated proteins through SUMO-interacting motifs (SIM). Notably, polySUMO chain formation is a function of SUMO E4 ligases that specifically adds multiple SUMOs to the target lysine of the desired substrate. In Arabidopsis, E4 ligases are represented by two members of PROTEIN INHIBITOR OF ACTIVATED STAT-like 1/2 (PIAL1 and 2). The STUBLs present a remarkable pathway for the regulation of protein turnover and demonstrate an important link between SUMOylation and ubiquitination.

Like ubiquitination, the SUMO-modification pathway is a three-step process catalyzed by an enzymatic cascade: E1 or SUMO-activating enzymes (SAE), E2 or SUMO-conjugating enzyme (SCE), and SUMO E3; which, respectively, activate, transfer, and conjugate SUMO to desired targets. The activation of SUMO is an ATP-dependent process and forms SUMO adenylate, releasing pyrophosphate. Further, SUMO is transferred to catalytic cysteine of E1 forming the E1-SUMO complex. Thio-esterification of SUMO to E1 is accompanied by a transesterification reaction, during which E2 is recruited to E1 UFD, which facilitates the subsequent transfer of SUMO to the internal cysteine of E2 enzymes. SUMO-conjugating enzymes are at the core of the SUMOylation pathway because they accomplish an essential link, directly or indirectly, between activated SUMO and its substrate *via* SUMO E3 ligases. In the former case, substrate selectivity is solely conferred by E2 enzymes, while the latter projects E3 ligases as a common interface which binds the charged SUMO~E2 complex and substrate, orienting them in a way that facilitates the discharge of SUMO from E2 to a relevant substrate. The ability of E3 ligases to hold SUMO in a closed conformation through SIM motifs helps the efficient transfer of activated SUMO to the substrate protein.

E1 is a heterodimer of SAE1/2, where SAE1 is a small subunit with two equipotent isoforms, SAE1a and SAE1b, in plants. SAE2 is a large subunit with three functional domains: the adenylation domain, an active site with conserved Cys residue, and the UFD domain. Its C-terminal contains nuclear localization signal (NLS). Although the presence of SAE1a/1b in plants has not been related to any functional significance but contains a conserved Arg_16_, which is the only residue that interacts with ATP in the adenylation domain (Lois, 2010). In plants, E1 enzymes offer selectivity to a certain extent by determining the specific SUMO isoforms that facilitate the entry to a particular SUMO pathway and regulate conjugation rates. A second non-canonical NLS at the C-terminus of SAE2 was also reported, which drives complete nuclear localization of SAE2, along with canonical NLS (Más et al., 2020). Proteolytic cleavage of second NLS restricts SAE2 within the cytosol. This novel PTM enables cytosolic SUMOylation and plays a definite role during seed development. The nuclear-cytosolic shuttling of E1 in plants suggests response mechanisms to various signals by modulating subcellular SUMOylation (Más et al., 2020). Arabidopsis has a single *AtSCE1a* gene while rice contains two E2s, *OsSCE1*, and *OsSCE3* with contrasting roles in drought stress.

Plants have three types of SUMO E3 ligases: the SAP AND MIZ1 DOMAIN-CONTAINING LIGASE (SIZs); the METHYL METHANESULFONATE-SENSITIVITY PROTEIN 21 (MMS21s); and the PROTEIN INHIBITOR OF ACTIVATED STAT-LIKE (PIALs). The most conserved domain of SUMO E3 ligases is SIZ/PIAL-RING (SP-RING), flanked by the SP C-terminal domain (SP-CTD). It activates the E2~SUMO thioester bond for subsequent transfer reaction. SP-RING selectively establishes interaction with E2, while SP-CTD interacts with SUMO in a SIM-like manner to hold SUMO in a closed conformation. SP-RING domain displays similarities with α/β fold of RING and U-box domain of Ub-E3 ligases. In addition, SIZ1 and PIAL1/2 harbor domains and sequence motifs that mediate protein-protein interactions (PPI). The Plant HomeoDomain (PHD) is unique to plant SUMO ligases. It is required for SUMO-modification of global transcription factors E3 (GTE3) and helps in SUMOylation activity of SIZ1. SUMO E3 ligases, particularly SIZ1 and PIAL1/2, also contain SUMO-binding motifs and NLS. SIM motif of PIAL1/2 is associated with polySUMO chain assembly. Plant SIZ1 also possesses a valine-proline CONSTITUTIVE PHOTOMORPHOGENESIS PROTEIN 1 (VP COP1) binding motif, through which it physically interacts with the substrate binding pocket of Ub E3 ligase COP1, which is a point of integration of SUMOylation and ubiquitination (Jmii and Cappadocia, 2021). SIZ1 ligases are highly pleiotropic regulating multitudes of plant processes related to growth, development, reproduction, phytohormone signaling, and stress responses. MMS21 has been majorly associated with DNA damage responses, cell cycle regulation, and regulation of 26S proteasome activity. PIAL1 and 2 regulate transcriptional silencing and salt stress responses. Interestingly, knockout mutants of *pial1* and *pial2* showed improved salt tolerance with better PSII activity and higher biomass and displayed a greener phenotype (Jmii and Cappadocia, 2021).

Although reversible SUMOylation machinery in plants is conserved, it has a variable number of enzyme proteoforms with plant-specific functions. The expansion of the SUMO system in plants suggests the acquisition of novel functions by the SUMO-modification system during evolution. Factors instrumental to the specificity of SUMOylation remain enigmatic because of limited information about the components. Our current understanding regarding the mechanism of SUMOylation relies largely on a single universally conserved E2 and countable E3 ligases. This contrasts with Ub-system, in which substrate selectivity and specificity are majorly conferred by diverse E2-E3 combinations. Unlike Ub E2 enzymes that strictly rely on E3 ligases for exquisite substrate selectivity, SUMO E2s are less reliant on E3 ligases. Rather, they can directly engage substrates with canonical SUMOylation motifs to direct the transfer of SUMO to a lysine residue in substrates. Nevertheless, SUMO ligases are essential for attaching SUMO to non-canonical motifs, thereby, expanding the substrate repertoire of SUMOylation (Gareau and Lima, 2010; Jmii and Cappadocia, 2021).

The cellular balance of SUMOylation and deSUMOylation is fine tuned by the action of E1-E2-E3 cascades and SUMO proteases, respectively. The downstream effect of SUMO-modification is, in part, mediated by effectors containing hydrophobic core possessing SUMO-interacting/binding motifs (SIM/SBM) surrounded by acidic and/or Ser residues. SIM/SBM harboring substrates through intramolecular interactions induce post-modification conformational changes. A non-covalent interaction between the modifier and its substrate adds specificity to the overall system since this interaction allows the substrate to select its cognate SUMO paralog. Functional consequences of SUMOylation imply critical PPIs that alter the conformation of modified target or change protein surface by exposing and/or masking interaction interfaces thereby facilitating or inhibiting trans-proteome interactions.

Signals that induce alterations in SUMOylome to regulate the structure, location, activity, solubility, stability, interaction profile, and PTM crosstalk of individual SUMO substrates allow reprogramming of cellular metabolism, signaling, gene expression, and chromatin remodeling to synchronize the physiological responses. It suggests the significance of SUMOylation in life-sustaining processes, such as cell division and proliferation, chromatin and epigenetic regulation, maintenance of nuclear integrity and transcription, differentiation and stemness, senescence, and stress responses.

### SUMOylation and abiotic stress responses

SUMO proteases have a vital role in regulating plant stress responses by balancing the cellular levels of SUMOylated and non-SUMOylated proteins. Mutational studies presented a paradigm that SUMOylation regulates multiple facets of plant biology predominantly through transcriptional regulation of gene expression. Arabidopsis mutants defective in SUMO1/2, SAE, and SCE showed embryonic lethality, while ligase- and protease-deficient mutants had physiological and developmental defects. For example, the mutated AtSIZ1, a SUMO Ligase, resulted in compromised tolerance to cold and drought, early flowering, and phosphate starvation symptoms (Ghimire et al., 2021). Hyper-SUMOylation under acute heat, cold, high salinity, drought, oxidative stress, and nutrient deficiency marks the conserved SUMO stress response (SSR) in plants (Miura et al., 2005; Catala et al., 2007; Chen et al., 2011; Ghimire et al., 2020; Roy and Sadanandom, 2021; Han et al., 2022). SIZ1-mediated SUMOylation positively responds to salt and drought-induced osmotic stress; enhances metal stress tolerance and light signaling; and regulates N, P, and ROS homeostasis in plants (Fang et al., 2022). SUMOylation regulates heat stress positively at transcriptional, post-transcriptional, and translational levels. Heat shock triggers SSR, which promotes mass movement of SUMO1/2 from the cytoplasm to the nucleus. Elevated temperature results in the accumulation of unfolded proteins, which requires activation of unfolded protein response (UPR). In Arabidopsis, under normal conditions, a co-chaperon in UPR, BAG7, and a TF, bZIP28, remains associated with AtBiP2 in the ER membrane. Heat-shock-induced accumulation of unfolded protein signals SUMOylation, BiP2 dissociation, and nuclear translocation of BAG7, where it associates with nuclear TF, WRKY29, to activate transcription of stress-responsive chaperons. At the same time, dissociated bZIP28 moves to the nucleus and causes induction of BiP3 gene expression by UPR. SCE1-mediated SUMOylation of DREB2A enhances its stability, which then binds to DRE-elements for inducing transcription of stress-responsive genes (Han et al., 2021, 2022) (Figure 4A). SUMO1 conjugation represses expression of heat-shock TF, AtHSFA2, during the recovery phase, and on second exposure to heat-shock, it gets deSUMOylated, inducing expression of heat-shock proteins. AtHSF2 SUMOylation and deSUMOylation provide a primary response to heat shock until the plant stabilizes its response and acquires thermotolerance (Cohen-Peer et al., 2010). Another aspect of plants' response to heat is thermomorphogenesis, during which plant undergoes elongation growth to enhance cooling effects. AtSIZ1-dependent SUMOylation of Ub-E3 ligase COP1 stimulates SIZ1 and HY5 degradation and PIF4 stabilization. While COP1 activity is essential for transducing high-temperature signals, SIZ1 amplifies stress signals through this feedback loop. Elevated PIF4 levels enhance thermomorphogenesis and inhibit SNC1-dependent autoimmunity. Thus, SIZ1 has mutually dependent roles in modulating growth and defense at elevated temperatures (Hammoudi et al., 2018).

Cold, salinity, and drought-related genes are mainly under transcriptional control of DREB TFs. DREB binds to DRE/CRT *cis-*elements to activate their target gene expression. Low temperature induces SUMOylation of ICE1 at K_393_. SUMO-ICE1 induced overexpression of DREB1/CBF3 leads to activation of CBF regulon and provides cold acclimatization. Also, SUMO-ICE1 counteracts the action of HOS1, which catalyzes polyubiquitination and proteasomal degradation of ICE1 (Figure 4B). Moreover, SUMO-conjugated ICE1 represses MYB15 (R_2_R_3_ MYB TF) expression, which, although induced by cold stress, is a negative regulator of CBF genes (Roy and Sadanandom, 2021). ICE1 is a pioneer of the cold signaling pathway in plants. ICE1-dependent induction of CBF expression involves interplay between GA and JA signaling. At optimal temperatures, JAZ, a repressor of JA signaling, remains associated with ICE1 to suppress CBF expression activation. However, low temperatures increase the ICE1 levels and activity through the accumulation of DELLA, the negative regulator of GA signaling, which binds JAZ to release ICE1. Consequently, the elevation in CBF gene expression reinforces DELLA accumulation, which, in turn, releases more JAZ-bound ICE1. Therefore, CBF3 and DELLA show mutual regulation to modulate plant growth under stress. CBF is an upstream transcriptional activator of cold-responsive (COR) genes. Studies report that MeJA also contributes to the upregulation of CBF signaling by degrading JAZ. Also, DELLA interacts with JAZ to uplift JAZ-mediated inhibition of MYC2 TF, which is a positive regulator of JA signaling and enhances CBF gene transcription by binding ICE1 (Zhou et al., 2017; Ritonga and Chen, 2020). Brassinosteroids also impart basal freezing tolerance, as well as cold acclimatization, without retarding the plant growth. BR-deficient mutants showed repression of 6% of COR genes majority, which all were CBF controlled. BIN2 phosphorylates CESTA (CES) to prevent its SUMOylation. CES, a close homolog of BRASSINOSTEROIDS ENHANCED EXPRESSION1 (BEE1) and BEE3, is a bHLH TF, whose abundance, activity, and subnuclear localization are BR-regulated. BR, upon binding with its receptor BRI1, activates BR signaling to repress BIN2 activity, promotes SUMOylation (K_72_), and translocates CES to the nucleus to induce COR gene expressions in CBF-dependent, as well as in CBF-independent, ways. In CBF-dependent expression of COR genes, CES has been shown to bind G-box motifs in CBF promoters (Eremina et al., 2016).

Srivastava et al. (2017) demonstrated the role of rice SUMO proteases (OsOTS1) in the mitigation of drought. OsOTS1 regulates the activity of OsbZIP23 and confers drought tolerance in an ABA-dependent manner. When sufficient hydration prevails, OsOTS1 interacts and maintains the deSUMOylated state of OsbZIP23. Drought-induced ABA accumulation promotes OsOTS1 degradation and OsbZIP23 SUMOylation, which positively regulates the expression of drought tolerant genes. OsOTS1 is also known to deSUMOylate and facilitate the degradation of DELLA proteins. Hyper-SUMOylation, followed by water-stress-mediated turnover of OTS1 proteases, results in the accumulation of rice DELLA proteins, SLR1, which impedes plant growth (Srivastava et al., 2017). OsSCE1 and SCE2 also contribute to drought tolerance but have opposing effects. Over-expression of OsSCE1 leads to hypersensitive phenotype, while OsSCE2 overexpression led to improved drought tolerance. In Arabidopsis, genome-wide expression analysis revealed that 1,700 genes are induced under desiccated conditions, and SIZ1 regulates the expression of ~300 drought-induced genes through DREB2A and ABA-independent pathways. Such genes encode enzymes of anthocyanin biosynthesis and jasmonate signaling. This is consistent with the role of anthocyanin in the ROS detoxification pathway (Catala et al., 2007). Epistatic relations between AtSIZ1 and AtOTS1/2 have been shown to modulate stomatal closure and osmotic stress responses in Arabidopsis (Castro et al., 2016). Mutants, defective in AtSIZ1, exhibit reduced stomatal aperture, improved drought tolerance, and ABA hypersensitivity. Stomatal closure-dependent drought resistance is an effect of SA-induced ROS production, while ABA hypersensitivity is due to inhibition of SIZ1-mediated ABI5 SUMOylation. In the former case, the phenotype was complemented by exogenous treatment with salicylhydroxamic acid (SHAM) and azide but the application of NADPH oxidase inhibitor had no effect. It was inferred that SA accumulation and peroxidase-catalyzed ROS production are responsible for the *siz1* phenotype. Thus, it can be concluded that SUMO E3 ligase, SIZ1, modulates endogenous SA levels to regulate stomatal closure and drought tolerance. Notably, these effects are independent of ABA. Also, SIZ1-mediated SUMOylation of ABI5 has negative effects on ABA signaling (Miura et al., 2013). Similar to SIZ1, MMS21 is a negative regulator of ABA-dependent drought responses. Recent studies on *Malus domestica* evidenced the implication of SUMOylation in regulating DREB2A stability as a critical determinant of drought tolerance. Interestingly, it was reported that drought tolerance in apples involves a complex network of increased and reduced levels of SUMOylation and ubiquitination, respectively, where SUMOylated MdDREB2A serves as a target for RNF4-mediated ubiquitination and degradation (Li et al., 2022). Pepper dehydration-responsive homeobox1 domain (CaDRHB1) TF serves as a positive regulator of the desiccation stress response. The stability of CaDRHB1 is enhanced by CaDRHB1-interacting SIZ1 (CaDSIZ1) mediated SUMOylation under drought conditions by positively regulating ABA signaling and drought tolerance (Joo et al., 2022). AUXIN RESPONSE FACTOR7 (ARF7) plays a central role in hydropatterning, regulated by SUMOylation and deSUMOylation to modulate lateral root development. On the airside of the root, lateral root growth is suppressed due to ARF7 SUMOylation that facilitates its interaction with indole-3-acetic acid repressor *via* SIM motifs. Consequently, it inhibits ARF7 transcriptional activity and, hence, leads to activation of downstream-acting auxin-responsive genes. Root exposed to water has denser lateral roots to aid the water uptake. This happens when ARF7 is maintained in a non-SUMOylated state, likely, by the action of OTS1 proteases that promotes transcriptional activation of ARF7 target genes to stimulate lateral root formation (Roy and Sadanandom, 2021).

Likewise, SUMOylation affects the plants' responses to salinity. This is mediated by modifying transcriptional activity of an R2R3-MY-type TF, MYB30, which is known to regulate several other developmental, hormonal, and stress signaling and is subject to multiple PTMs. For example, S-nitrosylation of MYB30 consequently hampers its DNA binding ability; its activity is switched off following an initial stress response. Here, AtSIZ1-mediated SUMOylation of MYB30 at K283 leads to the expression of Alternative oxidase 1a (AOX1a), which subsequently activates cyanide-resistant respiration to restore cellular redox homeostasis to provide salt tolerance (Gong et al., 2020). AtOTS1/2 proteases govern plant growth under salinity stress. Increased levels of SUMO1/2 conjugated certain target proteins are also critical for retarding growth to tolerate stress. Therefore, OTS1/2 not only enhances salt tolerance but also balances plant growth and survival under stressful conditions (Conti et al., 2009). Salt tolerance in rice involves homologs of Arabidopsis AtOTS1, but unlike AtOTS1, salt stress signals OsOTS1 degradation, which leads to accumulation of SUMO conjugates and eventually restraints growth through a common mechanism of SUMOylation-dependent abundance and stabilization of DELLA repressors. Salt resistance conferred by OsOTS1 is attributed to two interrelated mechanisms: regulation of gene-encoding components of the antioxidant system; and their activation by deSUMOylating enzymes, such as peroxidases and dismutases (Srivastava et al., 2016). SUMOylation also plays a significant role in nutrient acquisition and deficiency responses. Phosphorus, a macroelement, is available as inorganic phosphorous (Pi) in soil. Its low diffusion rates and fixation as organic complexes pose nutritional stress and affect the normal physiology of the plant. SUMOylation is instrumental in regulating the Pi homeostasis in plants. The SIZ1 is reported to control phosphate deficiency-induced responses in Arabidopsis and rice. The primary root tip in Arabidopsis can inherently sense local Pi concentrations irrespective of the internal Pi levels in the plant. Under Pi deficit condition, root tip cells experience progressive loss of meristematic activity, causing determinate root growth. This response was even aggravated in T-DNA insertional mutants of *siz1*, which also displayed lateral root development during Pi deprivation. SIZ1 was assumed to exert its regulatory influence on root system architecture in an auxin-dependent manner. Augmented growth of root hair number and length is also a well-characterized Pi-deficient adaptive response in Arabidopsis. The further analysis illustrated the negative impact of SIZ1 on auxin distribution patterns. Rice homolog of AtSIZ1, OsSIZ1, partially rescued the *siz1* mutant phenotype, while stimulated uptake of Pi observed in creeping bentgrass (*Agrostis stolonifera*) overexpressing OsSIZ1 suggests the possible implications of OsSIZ1 in the regulation of various root traits during Pi deficiency. Interestingly, Pi-deprived rice plants, having a more complex root system, showed significant stimulation in primary root growth and length of adventitious roots. Although auxin-signaling components, OsARF12 and OsARF16, are known to modulate Pi-deficiency-induced auxin-dependent root development responses, their dependence on SIZ1 is unexplored. These adaptive morphophysiological responses are the result of differential regulation of phosphate starvation responsive (PSR) gene expression by SIZ1. Some PSR genes like *Atpht1/4, AtRNS1*, and *AtIPS1* identified in Arabidopsis are shown to be involved in Pi acquisition, mobilization, and target mimicry, respectively. *AtG3Pp1* encodes for a putative organic P transporter whose expression is regulated by Pi availability in soil. SIZ1 induces the expression of *AtRNS1* and *AtIPS1* under Pi deficiency, while downregulating *Atpht1* and *AtG3Pp1* under optimal Pi concentrations, suggesting that SIZ1-dependent SUMOylation has stimulatory, as well as inhibitory, effects on the expression of PSR genes (Datta et al., 2018). Transcriptional activation of PSR genes is controlled by PHR1 (MYB TF), which binds to the PHR1-specific binding sequence (P1BS) found in the promoter region of PSR genes. SIZ1-mediated PHR1 SUMOylation at K_261_ and K_372_ residues stabilize the TF, resulting in increased abundance and DNA-binding activity of PHR1, thereby inducing target genes to exert adaptive changes in response to Pi starvation (Roy and Sadanandom, 2021). Rice PHR1-related TF, OsPHR2, is the major regulator of PSR genes and maintains cellular Pi homeostasis. OsSIZ1-modulated SUMOylation state of OsPHR1 drives the differential regulation of PSR genes under variable Pi regime (Datta et al., 2018). SUMOylation also facilitates coping with Fe deficiency. Differential regulation of stress-responsive genes through SUMOylation is mediated by the stabilization of upstream TFs. In apple, MdSIZ1 is known to directly SUMOylate and stabilize MdbHLP104 TF, which promotes Fe-responsive gene transcription. Resistance toward Al toxicity is acquired through activation of Al-resistance genes that are under transcriptional control of C_2_H_2_-type TF, STOP1. SUMOylation of STOP1 confers stability and stimulates the expression of target genes (Han et al., 2021). Excessive Cu^2+^ accumulation disrupts ROS homeostasis, which leads to toxicity. High Cu^2+^ level tolerance involves SUMOylation and deSUMOylation. To achieve optimal Cu^2+^ levels, plants precisely regulate the Cu^2+^ uptake, translocation, efflux, and sequestration. Related studies suggest that plant response to Cu^2+^ toxicity is a fine balance of SIZ1-mediated SUMOylation and OTS1-mediation deSUMOylation, which help plants to acquire Cu^2+^ homeostasis and tolerance (Zhan et al., 2018). SIZ1-mediated SUMOylation of NR NIA1 and NIA2 facilitate nitrogen assimilation, thereby, affecting overall growth and development in Arabidopsis (Datta et al., 2018).

## Conclusion and future perspectives

Every organism undergoes differential reprogramming in response to various stresses and generates appropriate responses at the biochemical/cellular/organ/organism level. Being the workhorses of the cell, proteome organization is the foremost system to get amended upon the perception of stress stimuli. Changes in membrane integrity, ion fluxes, and osmotic potential are the primary physiological disruptions observed under various stresses. To realize the stress type and generate the respective response, the cell implements exclusive and extensive rearrangements at omic (proteome, genome, transcriptome, proteome, ionome, interactome, etc.) levels. Early responses are pivotal to confer immediate-early protection and largely manifest rapid, transient, and dynamic changes in proteome architecture. This eventually stabilizes the otherwise repressed stress-responsive factors through active inhibition/activation/elimination of upstream regulators, which subsequently modulates gene expression profile to initiate long-term acclimation responses. The tolerance of plants to abiotic stress(es) is a complex phenomenon composite of manifold adaptive responses and synchronized operation of multiple signaling pathways simultaneously. A certain degree of overlap in the context of common elements that serve as PTM hotspots and function as switch boxes is crucial for such coordination and mediates the functional crosstalk in a stimulus-specific manner. Cross-modifications of catalytic machinery by distinct PTMs in the dialog are the alternative methods to mediate their crosstalk to respond to stress stimuli. Moreover, feedback mechanisms are also a part of the stress signaling network that regulates the intensity and duration of stress responses, thereby enabling plants to restore homeostasis.

We reviewed the role of ubiquitination and SUMOylation as covalent protein modifications in plants' immunity against abiotic stresses. Molecular mechanisms and enzymatic machineries underlying these modifications have been well-elucidated in eukaryotes, although prokaryotes have a UPS-like degradation system but lack SUMO proteins. The evolution of SUMOylation in eukaryotes suggests the expansion of ubiquitin-like modifications from a mere protein degradation unit to the regulators of virtually all aspects of protein functions. It is in agreement with the increasing complexity of eukaryotes. Although there is a striking similarity between ubiquitination and SUMOylation mechanism, as both are lysine residue modifiers and utilize concerted action of three enzyme systems, these PTMs often modify the same or different lysine residue(s) on single and/or multiple substrates and mutually regulate each other. It is quite likely that the dynamic addition of Ub and SUMO on the same protein might have opposing consequences, where the former usually accelerates the protein turnover, while the latter leads to stabilization, thus promoting the function of the target protein(s). This is not always the case; evidence supporting the complementary, as well as independent functions, has also been reported. For instance, (de)ubiquitination, apart from proteolysis, also codes for non-proteolytic functions during various developmental and stress signaling.

UPS has mainly evolved as a major protein degradation pathway in eukaryotes but underwent a massive gain-of-function and positive selection, committing ubiquitin-dependent processes to novel cellular functions. The conservation status of the ubiquitin modification system and its considerable expansion in higher organisms is consistent with its widespread role in regulating critical biological processes in the plant. SUMOylation evolved as a critical transcriptional modulator in plants due to its role in the regulation of a large complement of TFs and primarily controls their subcellular localization, stability, and interaction affecting chromatin association ability. Thus, group SUMOylation of multiple substrates facilitates their association to form functional protein complexes due to the enhanced SUMO-SIM interactions. Largely acting as a synergist or antagonist partner of ubiquitination, SUMOylation often cooperates or competes with the latter for similar targets to either support or defy each other's effect. STUBLs have emerged as exclusive crosstalk agents between SUMOylation and ubiquitination and specifically define substrates for the latter through SIM-mediated interpretation of SUMOylation on common substrates. Interestingly, STUBLs catalyzed SUMO-Ub hybrid chain formation is also reported to mark proteins as degrons. Ubiquitination and SUMOylation-mediated regulation of COP1 Ub ligase activity represent a classic example of their interdependent behavior. COP1 is a negative regulator of photomorphogenesis that ubiquitinates and signals positive regulators of photomorphogenesis for degradation in dark conditions. SIZ1 catalyzes SUMOylation of COP1 at K_193_ to enhance its trans-ubiquitination activity but has no effect on its abundance, dimerization, and translocation state, indicating that COP1 activity is only partly regulated by SIZ1. SUMOylation also facilitates the COP1 mediated degradation of ubiquitinated SIZ1, which suggests a positive regulation integrated negative feedback mechanism for mutually dependent functions of COP1 and SIZ1 ligases. Light-induced SUMOprotease-mediated deSUMOylation of COP1 and photoreceptor-mediated nuclear exclusion of COP1 lead to the stabilization of photomorphogenic factors, which activate light-responsive genes involved in photomorphogenic development in plants. Moreover, ubiquitination and SUMOylation cooperate with phosphorylation to form a tripartite regulatory network. This can be understood by their co-regulation of BR signaling where the transcription activator of BR responsive genes, BZR1, is targeted by all three PTMs to switch on and off BR responses under growth and stress conditions. BZR1 is phosphorylated by BIN2 for subsequent proteasomal degradation. SUMOylation counteracts the effect of phosphorylation and stabilizes BZR1 for activation of BR-responsive genes. However, salt stress-induced downregulation of BR responses is triggered by destabilization and accelerated degradation of phosphorylated BZR1. It is a consequence of the cytosolic accumulation of ULP1a/ELS1 SUMO protease, which deSUMOylates BZR1 and enhances its affinity for BIN2 phosphorylation and ubiquitin-dependent degradation (Han et al., 2021).

Ubiquitin and SUMOs, as well as their catalytic modules, have been considerably characterized, yet their potential substrates and downstream interacting partners are still enigmatic and need further investigations to decipher the molecular mechanisms. This will help to individually and/or holistically understand the operative signaling networks during various abiotic stresses to manifest abiotic stress tolerance in plants. This review will help to comprehend the importance of protein modifying enzymes as potential targets for bioengineering to accelerate the development of stress-tolerant plants in the future.

## Author contributions

DKY conceived and designed the study, supervised the study, and revised the final version of the manuscript. MS, AS, and NY performed the literature search and wrote the manuscript. AS and NY designed the graphics. All authors have approved the final manuscript for submission.

## Funding

This work was supported by the Department of Science and Technology, Science and Engineering Research Board, Government of India, under Start-up Research Grant (SB/YS/LS-185/2013) to DKY.

## Conflict of interest

The authors declare that the research was conducted in the absence of any commercial or financial relationships that could be construed as a potential conflict of interest.

## Publisher's note

All claims expressed in this article are solely those of the authors and do not necessarily represent those of their affiliated organizations, or those of the publisher, the editors and the reviewers. Any product that may be evaluated in this article, or claim that may be made by its manufacturer, is not guaranteed or endorsed by the publisher.

## References

[B1] Agarwal P. Agarwal P. K. Nair S. Sopory S. K. Reddy M. K. (2007). Stress-inducible DREB2A transcription factor from *Pennisetum glaucum* is a phosphoprotein and its phosphorylation negatively regulates its DNA-binding activity. Mol. Genet. Genomics 277, 189–198. 10.1007/s00438-006-0183-z 17089163

[B2] Alexandersson E. Fraysse L. Sjövall-Larsen S. Gustavsson S. Fellert M. Karlsson M.. (2005). Whole gene family expression and drought stress regulation of aquaporins. Plant Mol. Biol. 59, 469–484. 10.1007/s11103-005-0352-1 16235111

[B3] Al-Saharin R. Hellmann H. Mooney S. (2022). Plant E3 ligases and their role in abiotic stress response. Cells 11, 890. 10.3390/cells11050890 35269512 PMC8909703

[B4] Aquila L. Atanassov B. S. (2020). Regulation of histone ubiquitination in response to DNA double strand breaks. Cells 9, 1699. 10.3390/cells9071699 32708614 PMC7407225

[B5] Augustine R. C. Vierstra R. D. (2018). SUMOylation: re-wiring the plant nucleus during stress and development. Curr. Opin. Plant Biol. 45, 143–154. 10.1016/j.pbi.2018.06.006 30014889

[B6] Berndsen C. E. Wolberger C. (2014). New insights into ubiquitin E3 ligase mechanism. Nat. Struct. Mol. Biol. 21, 301–307. 10.1038/nsmb.2780 24699078

[B7] Bu Q. Li H. Zhao Q. Jiang H. Zhai Q. Zhang J.. (2009). The arabidopsis RING finger E3 ligase RHA2a is a novel positive regulator of abscisic acid signaling during seed germination and early seedling development. Plant Physiol. 150, 463–481. 10.1104/pp.109.135269 19286935 PMC2675735

[B8] Callis J. (2014). The ubiquitination machinery of the ubiquitin system. The Arabidopsis Book 12, e0174. 10.1199/tab.0174 25320573 PMC4196676

[B9] Cao Y. Dai Y. Cui S. Ma L. (2008). Histone H2B monoubiquitination in the chromatin of FLOWERING LOCUS C regulates flowering time in Arabidopsis. Plant Cell 20, 2586–2602. 10.1105/tpc.108.062760 18849490 PMC2590739

[B10] Castro P. H. Couto D. Freitas S. Verde N. Macho A. P. Huguet S.. (2016). SUMO proteases ULP1c and ULP1d are required for development and osmotic stress responses in *Arabidopsis thaliana*. Plant Mol. Biol. 92, 143–159. 10.1007/s11103-016-0500-9 27325215

[B11] Castro P. H. Tavares R. M. Bejarano E. R. Azevedo H. (2012). SUMO, a heavyweight player in plant abiotic stress responses. Cell. Mol. Life Sci. 69, 3269–3283. 10.1007/s00018-012-1094-2 22903295 PMC11114757

[B12] Catala R. Ouyang J. Abreu I. A. Hu Y. Seo H. Zhang X.. (2007). The Arabidopsis E3 SUMO ligase SIZ1 regulates plant growth and drought responses. Plant Cell 19, 2952–2966. 10.1105/tpc.106.049981 17905899 PMC2048692

[B13] Chen C. C. Chen Y. Y. Tang I. C. Liang H. M. Lai C. C. Chiou J. M.. (2011). Arabidopsis SUMO E3 Ligase SIZ1 is involved in excess copper tolerance. Plant Physiol. 156, 2225–2234. 10.1104/pp.111.178996 21632972 PMC3149952

[B14] Chen L. Hellmann H. (2013). Plant E3 ligases: flexible enzymes in a sessile world. Mol. Plant 6, 1388–1404. 10.1093/mp/sst005 23307436

[B15] Chinnusamy V. Ohta M. Kanrar S. Lee B. Hong X. Agarwal M.. (2003). ICE1: a regulator of cold-induced transcriptome and freezing tolerance in Arabidopsis. Genes Dev. 17, 1043–1054. 10.1101/gad.1077503 12672693 PMC196034

[B16] Christensen A. H. Sharrock R. A. Quail P. H. (1992). Maize polyubiquitin genes: structure, thermal perturbation of expression and transcript splicing, and promoter activity following transfer to protoplasts by electroporation. Plant Mol. Biol. 18, 675–689. 10.1007/BF00020010 1313711

[B17] Chung E. Cho C.-W. So H.-A. Kang J.-S. Chung Y. S. Lee J.-H. (2013). Overexpression of VrUBC1, a mung bean E2 ubiquitin-conjugating enzyme, enhances osmotic stress tolerance in Arabidopsis. PLoS One 8, e66056. 10.1371/journal.pone.0066056 23824688 PMC3688854

[B18] Cohen-Peer R. Schuster S. Meiri D. Breiman A. Avni A. (2010). Sumoylation of Arabidopsis heat shock factor A2 (HsfA2) modifies its activity during acquired thermotholerance. Plant Mol. Biol. 74, 33–45. 10.1007/s11103-010-9652-1 20521085

[B19] Collins G. A. Goldberg A. L. (2017). The logic of the 26S proteasome. Cell 169, 792–806. 10.1016/j.cell.2017.04.023 28525752 PMC5609836

[B20] Conti L. Kioumourtzoglou D. O.' Donnell E. Dominy P. Sadanandom A. (2009). OTS1 and OTS2 SUMO proteases link plant development and survival under salt stress. Plant Signal. Behav. 4, 225–227. 10.4161/psb.4.3.7867 19721757 PMC2652536

[B21] Conti L. Price G. O'Donnell E. Schwessinger B. Dominy P. Sadanandom A. (2008). Small ubiquitin-like modifier proteases OVERLY TOLERANT TO SALT1 and−2 regulate salt stress responses in Arabidopsis. Plant Cell 20, 2894–2908. 10.1105/tpc.108.058669 18849491 PMC2590731

[B22] Cui F. Liu L. Li Q. Yang C. Xie Q. (2012). UBC32 Mediated oxidative tolerance in Arabidopsis. J. Genet. Genomics 39, 415–417. 10.1016/j.jgg.2012.05.005 22884097

[B23] Datta M. Kaushik S. Jyoti A. Mathur N. Kothari S. L. Jain A.. (2018). SIZ1-mediated SUMOylation during phosphate homeostasis in plants: looking beyond the tip of the iceberg. Semin. Cell Dev. Biol. 74, 123–132. 10.1016/j.semcdb.2017.09.016 28903074

[B24] Deribe Y. L. Pawson T. Dikic I. (2010). Post-translational modifications in signal integration. Nat. Struct. Mol. Biol. 17, 666–672. 10.1038/nsmb.1842 20495563

[B25] Deshaies R. J. Joazeiro C. A. P. (2009). RING domain E3 ubiquitin ligases. Annu. Rev. Biochem. 78, 399–434. 10.1146/annurev.biochem.78.101807.093809 19489725

[B26] Dill A. Thomas S. G. Hu J. Steber C. M. Sun T. (2004). The Arabidopsis F-box protein SLEEPY1 targets gibberellin signaling repressors for gibberellin-induced degradation. The Plant Cell 16, 1392–1405. 10.1105/tpc.020958 15155881 PMC490034

[B27] Dong C.-H. Agarwal M. Zhang Y. Xie Q. Zhu J.-K. (2006). The negative regulator of plant cold responses, HOS1, is a RING E3 ligase that mediates the ubiquitination and degradation of ICE1. *Proc. Natl. Acad. Sci*. U. S. A. 103, 8281–8286. 10.1073/pnas.0602874103 16702557 PMC1472463

[B28] Dooki A. D. Mayer-Posner F. J. Askari H. Zaiee A. Salekdeh G. H. (2006). Proteomic responses of rice young panicles to salinity. Proteomics 6, 6498–6507. 10.1002/pmic.200600367 17163441

[B29] dos Reis S. P. Tavares Lde S. Costa Cde N. Brígida A. B. de Souza C. R. (2012). Molecular cloning and characterization of a novel RING zinc-finger protein gene upregulated under *in vitro* salt stress in cassava. Mol. Biol. Rep. 39, 6513–6519. 10.1007/s11033-012-1479-1 22307786

[B30] Dove K. K. Klevit R. E. (2017). RING-between-RING E3 ligases: emerging themes amid the variations. J Mol. Biol. 429, 3363–3375. 10.1016/j.jmb.2017.08.008 28827147 PMC5675740

[B31] Drabikowski K. Ferralli J. Kistowski M. Oledzki J. Dadlez M. Chiquet-Ehrismann R. (2018). Comprehensive list of SUMO targets in Caenorhabditis elegans and its implication for evolutionary conservation of SUMO signaling. Sci. Rep. 8, 1139. 10.1038/s41598-018-19424-9 29348603 PMC5773548

[B32] Ellis C. Turner J. G. Devoto A. (2002). Protein complexes mediate signalling in plant responses to hormones, light, sucrose and pathogens. Plant Mol. Biol. 50, 971–980. 10.1023/A:1021291522243 12516865

[B33] Eremina M. Unterholzner S. J. Rathnayake A. I. Castellanos M. Khan M. Kugler K. G.. (2016). Brassinosteroids participate in the control of basal and acquired freezing tolerance of plants. Proc. Natl. Acad. Sci. 113, E5982–E5991. 10.1073/pnas.1611477113 27655893 PMC5056081

[B34] Fang S. Hou X. Liang X. (2022). SIZ1-mediated SUMOylation responds to multiple abiotic stresses in plants. Environ. Exp. Bot. 201, 104977. 10.1016/j.envexpbot.2022.104977

[B35] Feng B. Li S. Wang Z. Cao F. Wang Z. Li G.. (2021). Systematic analysis of lysine 2- hydroxyisobutyrylation posttranslational modification in wheat leaves. PLoS ONE 16, e0253325. 10.1371/journal.pone.0253325 34138952 PMC8211214

[B36] Fernandez M. A. Belda-Palazon B. Julian J. Coego A. Lozano-Juste J. Iñigo S.. (2020). RBR-Type E3 Ligases and the ubiquitin-conjugating enzyme UBC26 regulate abscisic acid receptor levels and signaling. Plant Physiol. 182, 1723–1742. 10.1104/pp.19.00898 31699847 PMC7140949

[B37] Finkelstein R. R. Gampala S. S. L. Rock C. D. (2002). Abscisic acid signaling in seeds and seedlings. The Plant Cell 14, S15–S45. 10.1105/tpc.010441 12045268 PMC151246

[B38] Finkelstein R. R. Lynch T. J. (2000). The Arabidopsis abscisic acid response gene ABI5 encodes a basic leucine zipper transcription factor. Plant Cell 12, 599–609. 10.1105/tpc.12.4.599 10760247 PMC139856

[B39] Fukao T. Barrera-Figueroa B. E. Juntawong P. Peña-Castro J. M. (2019). Submergence and waterlogging stress in plants: aq review highlighting research opportunities and understudied aspects. Front. Plant Sci. 10, 340. 10.3389/fpls.2019.00340 30967888 PMC6439527

[B40] Fukao T. Yeung E. Bailey-Serres J. (2011). The submergence tolerance regulator SUB1A mediates crosstalk between submergence and drought tolerance in rice. Plant Cell 23, 412–427. 10.1105/tpc.110.080325 21239643 PMC3051255

[B41] Garbarino J. E. Rockhold D. R. Belknap W. R. (1992). Expression of stress-responsive ubiquitin genes in potato tubers. Plant Mol. Biol. 20, 235–244. 10.1007/BF00014491 1327270

[B42] García-Mauriño S. Monreal J. Alvarez R. Vidal J. Echevarría C. (2003). Characterization of salt stress-enhanced phosphoenolpyruvate carboxylase kinase activity in leaves of *Sorghum vulgare*: independence from osmotic stress, involvement of ion toxicity and significance of dark phosphorylation. Planta 216, 648–655. 10.1007/s00425-002-0893-3 12569407

[B43] Gareau J. R. Lima C. D. (2010). The SUMO pathway: emerging mechanisms that shape specificity, conjugation and recognition. Nat. Rev. Mol. Cell Biol. 11, 861–871. 10.1038/nrm3011 21102611 PMC3079294

[B44] Geiss-Friedlander R. Melchior F. (2007). Concepts in sumoylation: a decade on. Nat. Rev. Mol. Cell Biol. 8, 947–956. 10.1038/nrm2293 18000527

[B45] Genschik P. Parmentier Y. Durr A. Marbach J. Criqui M.-C. Jamet E.. (1992). Ubiquitin genes are differentially regulated in protoplast-derived cultures of *Nicotiana sylvestris* and in response to various stresses. Plant Mol. Biol. 20, 897–910. 10.1007/BF00027161 1281439

[B46] Ghimire S. Tang X. Liu W. Fu X. Zhang H. Zhang N.. (2021). SUMO conjugating enzyme: a vital player of SUMO pathway in plants. Physiol. Mol. Biol. Plants 27, 2421–2431. 10.1007/s12298-021-01075-2 34744375 PMC8526628

[B47] Ghimire S. Tang X. Zhang N. Liu W. Si H. (2020). SUMO and SUMOylation in plant abiotic stress. Plant Growth Regul. 91, 317–325. 10.1007/s10725-020-00624-1

[B48] Gong Q. Li S. Zheng Y. Duan H. Xiao F. Zhuang Y.. (2020). SUMOylation of MYB30 enhances salt tolerance by elevating alternative respiration via transcriptionally upregulating AOX1a in Arabidopsis. Plant J. 102, 1157–1171. 10.1111/tpj.14689 31951058

[B49] Guo Q. Zhang J. Gao Q. Xing S. Li F. Wang W. (2008). Drought tolerance through overexpression of monoubiquitin in transgenic tobacco. J. Plant Physiol. 165, 1745–1755. 10.1016/j.jplph.2007.10.002 18280007

[B50] Hammoudi V. Fokkens L. Beerens B. Vlachakis G. Chatterjee S. Arroyo-Mateos M.. (2018). The Arabidopsis SUMO E3 ligase SIZ1 mediates the temperature dependent trade-off between plant immunity and growth. PLOS Genet. 14, e1007157. 10.1371/journal.pgen.1007157 29357355 PMC5794169

[B51] Han D. Lai J. Yang C. (2021). SUMOylation: a critical transcription modulator in plant cells. Plant Sci. 310, 110987. 10.1016/j.plantsci.2021.110987 34315601

[B52] Han D. Yu Z. Lai J. Yang C. (2022). Post-translational modification: a strategic response to high temperature in plants. aBIOTECH 3, 49–64. 10.1007/s42994-021-00067-w PMC9590526 36304199

[B53] Hanada K. Zou C. Lehti-Shiu M. D. Shinozaki K. Shiu S.-H. (2008). Importance of lineage-specific expansion of plant tandem duplicates in the adaptive response to environmental stimuli. Plant Physiol. 148, 993–1003. 10.1104/pp.108.122457 18715958 PMC2556807

[B54] Hattori T. Totsuka M. Hobo T. Kagaya Y. Yamamoto-Toyoda A. (2002). Experimentally determined sequence requirement of ACGT-containing abscisic acid response element. Plant Cell Physiol. 43, 136–140. 10.1093/pcp/pcf014 11828032

[B55] Herrmann J. Lerman L. O. Lerman A. (2007). Ubiquitin and ubiquitin-like proteins in protein regulation. Circ. Res. 100, 1276–1291. 10.1161/01.RES.0000264500.11888.f0 17495234

[B56] Hicke L. (2001). Protein regulation by monoubiquitin. Nat. Rev. Mol. Cell Biol. 2, 195–201. 10.1038/35056583 11265249

[B57] Himmelbach A. Yang Y. Grill E. (2003). Relay and control of abscisic acid signaling. Curr. Opin. Plant Biol. 6, 470–479. 10.1016/S1369-5266(03)00090-6 12972048

[B58] Hotton S. K. Callis J. (2008). Regulation of cullin RING ligases. Annu. Rev. Plant Biol. 59, 467–489. 10.1146/annurev.arplant.58.032806.104011 18444905

[B59] Hu X. Xiao X. Zhang C. L. Wang G. L. Zhang Y. L. Li Y. Y.. (2022). Organization and regulation of the apple SUMOylation system under salt and ABA. Plant Physiol. Biochem. 182, 22–35. 10.1016/j.plaphy.2022.03.034 35460932

[B60] Hu Z. Song N. Zheng M. Liu X. Liu Z. Xing J.. (2015). Histone acetyltransferase GCN5 is essential for heat stress-responsive gene activation and thermotolerance in Arabidopsis. Plant J. 84, 1178–1191. 10.1111/tpj.13076 26576681

[B61] Hua Z. Vierstra R. D. (2011). The cullin-RING ubiquitin-protein ligases. Ann. Rev. Plant Biol. 62, 299–334. 10.1146/annurev-arplant-042809-112256 21370976

[B62] Huang J. Luo Z. Ying W. Cao Q. Huang H. Dong J.. (2017). 2-Hydroxyisobutyrylation on histone H4K8 is regulated by glucose homeostasis in *Saccharomyces cerevisiae*. Proc. Natl. Acad. Sci. U. S. A. 114, 8782–8787. 10.1073/pnas.1700796114 28768809 PMC5565412

[B63] Huibregtse J. M. Scheffner M. Beaudenon S. Howley P. M. (1995). A family of proteins structurally and functionally related to the E6-AP ubiquitin-protein ligase. *Proc. Natl. Acad. Sci*. U. S. A. 92, 2563–2567. 10.1073/pnas.92.7.2563 7708685 PMC42258

[B64] Jang J. Y. Kim D. G. Kim Y. O. Kim J. S. Kang H. (2004). An expression analysis of a gene family encoding plasma membrane aquaporins in response to abiotic stresses in *Arabidopsis thaliana*. Plant Mol. Biol. 54, 713–725. 10.1023/B:PLAN.0000040900.61345.a6 15356390

[B65] Jisha V. Dampanaboina L. Vadassery J. Mithöfer A. Kappara S. Ramanan R. (2015). Overexpression of an AP2/ERF type transcription factor OsEREBP1 confers biotic and abiotic stress tolerance in rice. PLoS ONE 10, e0127831. 10.1371/journal.pone.0127831 26035591 PMC4452794

[B66] Jmii S. Cappadocia L. (2021). Plant SUMO E3 ligases: function, structural organization, and connection with DNA. Front. Plant Sci. 12, 652170. 10.3389/fpls.2021.652170 33897743 PMC8064691

[B67] Joo H. Lim C. W. Lee S. C. (2022). Pepper SUMO E3 ligase CaDSIZ1 enhances drought tolerance by stabilizing the transcription factor CaDRHB1. New Phytol. 235, 2313–30. 10.1111/nph.18300 35672943

[B68] Kang H. Zhang M. Zhou S. Guo Q. Chen F. Wu J.. (2016). Overexpression of wheat ubiquitin gene, Ta-Ub2, improves abiotic stress tolerance of *Brachypodium distachyon*. Plant Sci. Int. J. Exp. Plant Biol. 248, 102–115. 10.1016/j.plantsci.2016.04.015 27181952

[B69] Kant S. Bi Y. M. Rothstein S. J. (2011). Understanding plant response to nitrogen limitation for the improvement of crop nitrogen use efficiency. J. Exp. Bot. 62, 1499–509. 10.1093/jxb/erq297 20926552

[B70] Kim D. Y. Scalf M. Smith L. M. Vierstra R. D. (2013). Advanced proteomic analyses yield a deep catalog of ubiquitylation targets in Arabidopsis. Plant Cell 25, 1523–1540. 10.1105/tpc.112.108613 23667124 PMC3694690

[B71] Kim H. T. Pyo K. Lledias F. Kisselev A. F. Scaglione K. M. Skowyra D.. (2007). Certain pairs of ubiquitin-conjugating enzymes (E2s) and ubiquitin-protein ligases (E3s) synthesize nondegradable forked ubiquitin chains containing all possible isopeptide linkages. J. Biol. Chem. 282, 17375–17386. 10.1074/jbc.M609659200 17426036

[B72] Kim J. Y. Park B. S. Park S. W. Lee H. Y. Song J. T. Seo H. S. (2018). Nitrate reductases are relocalized to the nucleus by atsiz1 and their levels are negatively regulated by COP1 and ammonium. Int. J. Mol. Sci. 19, 1202. 10.3390/ijms19041202 29662028 PMC5979280

[B73] Kim S. Park S. Kwon H. Cho M. H. Kim B.-G. Chung J. H.. (2022). The rice abscisic acid-responsive RING finger E3 ligase OsRF1 targets OsPP2C09 for degradation and confers drought and salinity tolerance in rice. Front. Plant Sci. 12, 797940. 10.3389/fpls.2021.797940 35095969 PMC8792764

[B74] Kirkin V. McEwan D. G. Novak I. Dikic I. (2009). A role for ubiquitin in selective autophagy. Mol. Cell 34, 259–269. 10.1016/j.molcel.2009.04.026 19450525

[B75] Kirkpatrick D. S. Hathaway N. A. Hanna J. Elsasser S. Rush J. Finley D.. (2006). Quantitative analysis of *in vitro* ubiquitinated cyclin B1 reveals complex chain topology. Nat. Cell Biol. 8, 700–710. 10.1038/ncb1436 16799550

[B76] Ko J.-H. Yang S. H. Han K.-H. (2006). Upregulation of an Arabidopsis RING-H2 gene, XERICO, confers drought tolerance through increased abscisic acid biosynthesis. Plant J. 47, 343–355. 10.1111/j.1365-313X.2006.02782.x 16792696

[B77] Komander D. Clague M. J. Urbé S. (2009). Breaking the chains: structure and function of the deubiquitinases. Nat. Rev. Mol. Cell Biol. 10, 550–563. 10.1038/nrm2731 19626045

[B78] Komander D. Rape M. (2012). The ubiquitin code. Annu. Rev. Biochem. 81, 203–229. 10.1146/annurev-biochem-060310-170328 22524316

[B79] Kosová K. Vítámvás P. Prášil I. T. Renaut J. (2011). Plant proteome changes under abiotic stress - contribution of proteomics studies to understanding plant stress response. J. Proteomics 74, 1301–1322. 10.1016/j.jprot.2011.02.006 21329772

[B80] Kraft E. Stone S. L. Ma L. Su N. Gao Y. Lau O.-S.. (2005). Genome analysis and functional characterization of the E2 and RING-Type E3 ligase ubiquitination enzymes of Arabidopsis. Plant Physiol. 139, 1597–1611. 10.1104/pp.105.067983 16339806 PMC1310545

[B81] Kurepa J. Toh-E A Smalle J. (2008). 26S proteasome regulatory particle mutants have increased oxidative stress tolerance. Plant J. 53, 102–114. 10.1111/j.1365-313X.2007.03322.x 17971041

[B82] Kurepa J. Walker J. M. Smalle J. Gosink M. M. Davis S. J. Durham T. L.. (2003). The small ubiquitin-like modifier (SUMO) protein modification system in Arabidopsis. Accumulation of SUMO1 and−2 conjugates is increased by stress. J. Biol. Chem. 278, 6862–6872. 10.1074/jbc.M209694200 12482876

[B83] Lam Y. A. Xu W. De Martino G. N. Cohen R. E. (1997). Editing of ubiquitin conjugates by an isopeptidase in the 26S proteasome. Nature 385, 737–740. 10.1038/385737a0 9034192

[B84] Lee H. K. Cho S. K. Son O. Xu Z. Hwang I. Kim W. T. (2009). Drought stress-induced Rma1H1, a RING membrane-anchor E3 ubiquitin ligase homolog, regulates aquaporin levels via ubiquitination in transgenic Arabidopsis plants. Plant Cell 21, 622–641. 10.1105/tpc.108.061994 19234086 PMC2660634

[B85] Lee S.-J. Choi J.-Y. Sung Y.-M. Park H. Rhim H. Kang S. (2001). E3 ligase activity of RING finger proteins that interact with Hip-2, a human ubiquitin-conjugating enzyme. FEBS Lett. 503, 61–64. 10.1016/S0014-5793(01)02689-8 11513855

[B86] Lehti-shiu M. D. Shiu S. (2012). Diversity, classification and function of the plant protein kinase superfamily. Philos. Trans. R. Soc. Lond., B Biol. Sci. 367, 2619–2639. 10.1098/rstb.2012.0003 22889912 PMC3415837

[B87] Li H. Jiang H. Bu Q. Zhao Q. Sun J. Xie Q.. (2011). The Arabidopsis RING finger E3 ligase RHA2b acts additively with RHA2a in regulating abscisic acid signaling and drought response. Plant Physiol. 156, 550–563. 10.1104/pp.111.176214 21478367 PMC3177258

[B88] Li Q. Wang W. Wang W. Zhang G. Liu Y. Wang Y.. (2018). Wheat F-box protein gene TaFBA1 is involved in plant tolerance to heat stress. Front. Plant Sci. 9, 521. 10.3389/fpls.2018.00521 29740462 PMC5928245

[B89] Li S. He X. Gao Y. Zhou C. Chiang V. L. Li W. (2021). Histone acetylation changes in plant response to drought stress. Genes 12, 1409. 10.3390/genes12091409 34573391 PMC8468061

[B90] Li W. Bengtson M. H. Ulbrich A. Matsuda A. Reddy V. A. Orth A.. (2008). Genome-wide and functional annotation of human E3 ubiquitin ligases identifies MULAN, a mitochondrial E3 that regulates the organelle's dynamics and signaling. PLoS ONE 3, e1487. 10.1371/journal.pone.0001487 18213395 PMC2198940

[B91] Li W. Schmidt W. (2010). A lysine-63-linked ubiquitin chain-forming conjugase, UBC13, promotes the developmental responses to iron deficiency in Arabidopsis roots. Plant J. 62, 330–343. 10.1111/j.1365-313X.2010.04150.x 20113438

[B92] Li X. Zhou S. Liu Z. Lu L. Dang H. Li H.. (2022). Fine-tuning of SUMOylation modulates drought tolerance of apple. Plant Biotechnol. J. 20, 903–919. 10.1111/pbi.13772 34978131 PMC9055824

[B93] Lim S. D. Oh D. G. Park Y. C. Jang C. S. (2020). Molecular characterization of a RING E3 ligase SbHCI1 in sorghum under heat and abscisic acid stress. Planta 252, 89. 10.1007/s00425-020-03469-0 33064214

[B94] Liu B. Jiang Y. Tang H. Tong S. Lou S. Shao C.. (2021). The ubiquitin E3 ligase SR1 modulates the submergence response by degrading phosphorylated WRKY33 in Arabidopsis. Plant Cell 33, 1771–1789. 10.1093/plcell/koab062 33616649 PMC8254483

[B95] Liu Y. Zhu J. Sun S. Cui F. Han Y. Peng Z.. (2019). Defining the function of SUMO system in pod development and abiotic stresses in Peanut. BMC Plant Biol. 19, 593. 10.1186/s12870-019-2136-9 31884953 PMC7194008

[B96] Livneh I. Cohen-Kaplan V. Cohen-Rosenzweig C. Avni N. Ciechanover A. (2016). The life cycle of the 26S proteasome: from birth, through regulation and function, and onto its death. Cell Res.26, 869–885. 10.1038/cr.2016.86 27444871 PMC4973335

[B97] Lois L. M. (2010). Diversity of the SUMOylation machinery in plants. Biochem. Soc. Trans. 38, 60–64. 10.1042/BST0380060 20074036

[B98] Lopez-Molina L. Mongrand S. Chua N.-H. (2001). A postgermination developmental arrest checkpoint is mediated by abscisic acid and requires the ABI5 transcription factor in Arabidopsis. *Proc. Natl. Acad. Sci*. U. S. A. 98, 4782–4787. 10.1073/pnas.081594298 11287670 PMC31911

[B99] Lopez-Molina L. Mongrand S. Kinoshita N. Chua N.-H. (2003). AFP is a novel negative regulator of ABA signaling that promotes ABI5 protein degradation. Genes Dev. 17, 410–418. 10.1101/gad.1055803 12569131 PMC195991

[B100] Lopez-Molina L. Mongrand S. McLachlin D. T. Chait B. T. Chua N.-H. (2002). ABI5 acts downstream of ABI3 to execute an ABA-dependent growth arrest during germination. Plant J. 32, 317–328. 10.1046/j.1365-313X.2002.01430.x 12410810

[B101] Lorenz S. (2018). Structural mechanisms of HECT-type ubiquitin ligases. Biol. Chem. 399, 127–145. 10.1515/hsz-2017-0184 29016349

[B102] Lyzenga W. J. Stone S. L. (2012). Abiotic stress tolerance mediated by protein ubiquitination. J. Exp. Bot. 63, 599–616. 10.1093/jxb/err310 22016431

[B103] Maekawa S. Sato T. Asada Y. Yasuda S. Yoshida M. Chiba Y.. (2012). The Arabidopsis ubiquitin ligases ATL31 and ATL6 control the defense response as well as the carbon/nitrogen response. Plant Mol. Biol. 79, 217–227. 10.1007/s11103-012-9907-0 22481162

[B104] Marín I. (2010). Diversification and specialization of plant RBR ubiquitin ligases. PLoS ONE 5, e11579. 10.1371/journal.pone.0011579 20644651 PMC2904391

[B105] Marshall R. S. Vierstra R. D. (2019). Dynamic regulation of the 26S proteasome: from synthesis to degradation. Front. Mol. Biosci. 6, 40. 10.3389/fmolb.2019.00040 31231659 PMC6568242

[B106] Más A. Castaño-Miquel L. Carretero-Paulet L. Colomé N. Canals F. Lois L. M. (2020). Evolution of molecular determinants for SUMO-activating enzyme subcellular localization in plants. bioRxiv. 10.1101/2020.10.05.326249

[B107] Mattiroli F. Penengo L. (2021). Histone ubiquitination: an integrative signaling platform in genome stability. Trends Genet. 37, 566–581. 10.1016/j.tig.2020.12.005 33485674

[B108] Mazzucotelli E. Belloni S. Marone D. De Leonardis A. Guerra D. Di Fonzo N.. (2006). The E3 ubiquitin ligase gene family in plants: regulation by degradation. Curr. Genomics 7, 509–522. 10.2174/138920206779315728 18369404 PMC2269001

[B109] Mazzucotelli E. Mastrangelo A. M. Crosatti C. Guerra D. Stanca A. M. Cattivelli L. (2008). Abiotic stress response in plants: when post-transcriptional and post-translational regulations control transcription. Plant Sci. 174, 420–431. 10.1016/j.plantsci.2008.02.00530477211

[B110] Miricescu A. Goslin K. Graciet E. (2018). Ubiquitylation in plants: signaling hub for the integration of environmental signals. J. Exp. Bot. 69, 4511–4527. 10.1093/jxb/ery165 29726957

[B111] Miura K. Jin J. B. Haegawa P. M. (2007). Sumoylation, a post-translational regulatory process in plants. Curr. Opin. Plant Biol. 10, 495–502. 10.1016/j.pbi.2007.07.002 17720613

[B112] Miura K. Okamoto H. Okuma E. Shiba H. Kamada H. Hasegawa P. M.. (2013). SIZ1 deficiency causes reduced stomatal aperture and enhanced drought tolerance via controlling salicylic acid-induced accumulation of reactive oxygen species in Arabidopsis. Plant J. 73, 91–104. 10.1111/tpj.12014 22963672

[B113] Miura K. Rus A. Sharkhuu A. Yokoi S. Karthikeyan A. S. Raghothama K. G.. (2005). The Arabidopsis SUMO E3 ligase SIZ1 controls phosphate deficiency responses. Proc. Natl. Acad. Sci. U. S. A. 102, 7760–7765. 10.1073/pnas.0500778102 15894620 PMC1140425

[B114] Molinier J. Lechner E. Dumbliauskas E. Genschik P. (2008). Regulation and role of Arabidopsis CUL4-DDB1A-DDB2 in maintaining genome integrity upon UV stress. PLoS Genet. 4, e1000093. 10.1371/journal.pgen.1000093 18551167 PMC2396500

[B115] Morrell R. Sadanandom A. (2019). Dealing with stress: a review of plant SUMO proteases. Front. Plant Sci. 10:1122. 10.3389/fpls.2019.01122 31620153 PMC6759571

[B116] Narusaka Y. Nakashima K. Shinwari Z. K. Sakuma Y. Furihata T. Abe H.. (2003). Interaction between two cis-acting elements, ABRE and DRE, in ABA-dependent expression of Arabidopsis rd29A gene in response to dehydration and high-salinity stresses. Plant J. 34, 137–148. 10.1046/j.1365-313X.2003.01708.x 12694590

[B117] Ning Y. Jantasuriyarat C. Zhao Q. Zhang H. Chen S. Liu J.. (2011). The SINA E3 ligase OsDIS1 negatively regulates drought response in rice. Plant Physiol. 157, 242–255. 10.1104/pp.111.180893 21719639 PMC3165873

[B118] Pandey A. Chakraborty S. Datta A. Chakraborty N. (2008). Proteomics approach to identify dehydration responsive nuclear proteins from chickpea (*Cicer arietinum* L.). Mol. Cell. Proteomics 7, 88–107. 10.1074/mcp.M700314-MCP200 17921517

[B119] Park B. S. Song J. T. Seo H. S. (2011). Arabidopsis nitrate reductase activity is stimulated by the E3 SUMO ligase AtSIZ1. Nat. Commun. 2, 400. 10.1038/ncomms1408 21772271 PMC3160146

[B120] Park J.-A. Cho S. K. Kim J. E. Chung H. S. Hong J.-P. Hwang B.. (2003). Isolation of cDNAs differentially expressed in response to drought stress and characterization of the Ca-LEAL1 gene encoding a new family of atypical LEA-like protein homologue in hot pepper (*Capsicum annuum* L. cv. Pukang). Plant Sci. 165, 471–481. 10.1016/S0168-9452(03)00165-1

[B121] Peng L. Wan X. Huang K. Pei L. Xiong J. Li X.. (2019). AtPUB48 E3 ligase plays a crucial role in the thermotolerance of Arabidopsis. Biochem. Biophys. Res. Commun. 509, 281–286. 10.1016/j.bbrc.2018.12.123 30591216

[B122] Peng M. Hannam C. Gu H. Bi Y.-M. Rothstein S. J. (2007). A mutation in NLA, which encodes a RING-type ubiquitin ligase, disrupts the adaptability of Arabidopsis to nitrogen limitation. Plant J. 50, 320–337. 10.1111/j.1365-313X.2007.03050.x 17355433

[B123] Peth A. Uchiki T. Goldberg A. L. (2010). ATP-dependent steps in the binding of ubiquitin conjugates to the 26S proteasome that commit to degradation. Mol. Cell. 40, 671–681. 10.1016/j.molcel.2010.11.002 21095592 PMC3038635

[B124] Pickart C. M. Fushman D. (2004). Polyubiquitin chains: polymeric protein signals. Curr. Opin. Chem. Biol. 8, 610–616. 10.1016/j.cbpa.2004.09.009 15556404

[B125] Prakash S. Tian L. Ratliff K. S. Lehotzky R. E. Matouschek A. (2004). An unstructured initiation site is required for efficient proteasome-mediated degradation. Nat. Struct. Mol. Biol. 11, 830–837. 10.1038/nsmb814 15311270

[B126] Qin F. Sakuma Y. Tran L.-S. P. Maruyama K. Kidokoro S. Fujita Y.. (2008). Arabidopsis DREB2A-interacting proteins function as RING E3 ligases and negatively regulate plant drought stress–responsive gene expression. Plant Cell 20, 1693–1707. 10.1105/tpc.107.057380 18552202 PMC2483357

[B127] Ritonga F. N. Chen S. (2020). Physiological and molecular mechanism involved in cold stress tolerance in plants. Plants 9, 560. 10.3390/plants9050560 32353940 PMC7284489

[B128] Rittinger K. Ikeda F. (2017). Linear ubiquitin chains: enzymes, mechanisms and biology. Open Biol. 7, 170026. 10.1098/rsob.170026 28446710 PMC5413910

[B129] Rodrigo-Brenni M. C. Foster S. A. Morgan D. O. (2010). Catalysis of lysine 48-specific ubiquitin chain assembly by residues in E2 and ubiquitin. Mol. Cell 39, 548–559. 10.1016/j.molcel.2010.07.027 20797627 PMC2929935

[B130] Roy D. Sadanandom A. (2021). SUMO mediated regulation of transcription factors as a mechanism for transducing environmental cues into cellular signaling in plants. Cell. Mol. Life Sci. 78, 2641–2664. 10.1007/s00018-020-03723-4 33452901 PMC8004507

[B131] Ryu M. Y. Cho S. K. Kim W. T. (2010). The Arabidopsis C3H2C3-Type RING E3 ubiquitin ligase AtAIRP1 is a positive regulator of an abscisic acid-dependent response to drought Stress. Plant Physiol. 154, 1983–1997. 10.1104/pp.110.164749 20884812 PMC2996028

[B132] Sadanandom A. Bailey M. Ewan R. Lee J. Nelis S. (2012). The ubiquitin–proteasome system: central modifier of plant signalling. New Phytol. 196, 13–28. 10.1111/j.1469-8137.2012.04266.x 22897362

[B133] Saeki Y. Kudo T. Sone T. Kikuchi Y. Yokosawa H. Toh-e A.. (2009). Lysine 63-linked polyubiquitin chain may serve as a targeting signal for the 26S proteasome. EMBO J. 28, 359–371. 10.1038/emboj.2008.305 19153599 PMC2646160

[B134] Sakuma Y. Maruyama K. Osakabe Y. Qin F. Seki M. Shinozaki K.. (2006a). Functional analysis of an Arabidopsis transcription factor, DREB2A, involved in drought-responsive gene expression. Plant Cell 18, 1292–1309. 10.1105/tpc.105.035881 16617101 PMC1456870

[B135] Sakuma Y. Maruyama K. Qin F. Osakabe Y. Shinozaki K. Yamaguchi-Shinozaki K. (2006b). Dual function of an Arabidopsis transcription factor DREB2A in water-stress-responsive and heat-stress-responsive gene expression. *Proc. Natl. Acad. Sci*. U. S. A. 103, 18822–18827. 10.1073/pnas.0605639103 17030801 PMC1693746

[B136] Sato T. Maekawa S. Yasuda S. Domeki Y. Sueyoshi K. Fujiwara M.. (2011). Identification of 14-3-3 proteins as a target of ATL31 ubiquitin ligase, a regulator of the C/N response in Arabidopsis. Plant J. 68, 137–146. 10.1111/j.1365-313X.2011.04673.x 21668537

[B137] Sato T. Maekawa S. Yasuda S. Sonoda Y. Katoh E. Ichikawa T.. (2009). CNI1/ATL31, a RING-type ubiquitin ligase that functions in the carbon/nitrogen response for growth phase transition in Arabidopsis seedlings. Plant J. 60, 852–864. 10.1111/j.1365-313X.2009.04006.x 19702666

[B138] Schwechheimer C. Calderon Villalobos L. I. (2004). Cullin-containing E3 ubiquitin ligases in plant development. Curr. Opin. Plant Biol. 7, 677–686. 10.1016/j.pbi.2004.09.009 15491916

[B139] Sharma H. Batra R. Kumar S. Kumar M. Kumar S. Balyan H. S.. (2022). Identification and characterization of 20S proteasome genes and their relevance to heat/drought tolerance in bread wheat. Gene Rep. 27, 101552. 10.1016/j.genrep.2022.101552

[B140] Sharma M. Pandey G. K. (2019). OsPUB75, an Armadillo/U-box protein interacts with GSK3 kinase and functions as negative regulator of abiotic stress responses. Environ. Exp. Bot. 161, 388–398. 10.1016/j.envexpbot.2018.10.022

[B141] Shu K. Yang W. (2017). E3 ubiquitin ligases: ubiquitous actors in plant development and abiotic stress responses. Plant Cell Physiol. 58, 1461–1476. 10.1093/pcp/pcx071 28541504 PMC5914405

[B142] Shumyantseva V. V. Suprun E. V. Bulko T. V. Archakov A. I. (2014). Electrochemical methods for detection of post-translational modifications of proteins. Biosens. Bioelectron. 61, 131–139. 10.1016/j.bios.2014.05.001 24874656

[B143] Smalle J. Kurepa J. Yang P. Emborg T. J. Babiychuk E. Kushnir S.. (2003). The pleiotropic role of the 26S proteasome subunit RPN10 in Arabidopsis growth and development supports a substrate-specific function in abscisic acid signaling. Plant Cell 15:965–80. 10.1105/tpc.009217 12671091 PMC152342

[B144] Smalle J. Vierstra R. D. (2004). The ubiquitin 26s proteasome proteolytic pathway. Annu. Rev. Plant Biol. 55, 555–590. 10.1146/annurev.arplant.55.031903.141801 15377232

[B145] Smit J. J. Sixma T. K. (2014). ‘RBR E3-ligases at work. EMBO Rep. 15, 142–154. 10.1002/embr.201338166 24469331 PMC3989860

[B146] Srivastava A. K. Zhang C. Caine R. S. Gray J. Sadanandom A. (2017). Rice SUMO protease overly tolerant to salt 1 targets the transcription factor, OsbZIP23 to promote drought tolerance in rice. Plant J. 92, 1031–1043. 10.1111/tpj.13739 29024118

[B147] Srivastava A. K. Zhang C. Yates G. Bailey M. Brown A. Sadanandom A. (2016). SUMO is a critical regulator of salt stress responses in rice. Plant Physiol. 170, 2378–2391. 10.1104/pp.15.01530 26869703 PMC4825142

[B148] Srivastava M. Sadanandom A. (2020). An insight into the factors influencing specificity of the SUMO system in plants. Plants 9, 1788. 10.3390/plants9121788 33348543 PMC7767294

[B149] Stone S. L. (2014). The role of ubiquitin and the 26S proteasome in plant abiotic stress signaling. Front. Plant Sci. 5, 135. 10.3389/fpls.2014.00135 24795732 PMC3997020

[B150] Stone S. L. Hauksdóttir H. Troy A. Herschleb J. Kraft E. Callis J. (2005). Functional analysis of the RING-type ubiquitin ligase family of Arabidopsis. Plant Physiol. 137, 13–30. 10.1104/pp.104.052423 15644464 PMC548835

[B151] Stone S. L. Williams L. A. Farmer L. M. Vierstra R. D. Callis J. (2007). KEEP ON GOING, a RING E3 ligase essential for Arabidopsis growth and development, is involved in abscisic acid signaling. Plant Cell 18, 3415–3428. 10.1105/tpc.106.046532 17194765 PMC1785414

[B152] Sun C.-W. Callis J. (1997). Independent modulation of Arabidopsis thaliana polyubiquitin mRNAs in different organs and in response to environmental changes. Plant J. 11, 1017–1027. 10.1046/j.1365-313X.1997.11051017.x 9193073

[B153] Tamang B. G. Fukao T. (2015). Plant adaptation to multiple stresses during submergence and following desubmergence. Int. J. Mol. Sci. 16, 30164–30180. 10.3390/ijms161226226 26694376 PMC4691168

[B154] Teramura H. Yamada K. Ito K. Kasahara K. Kikuchi T. Kioka N.. (2021). Characterization of novel SUMO family genes in the rice genome. Genes Genet. Syst. 96, 25–32. 10.1266/ggs.20-00034 33731501

[B155] Tian F. Gong J. Zhang J. Feng Y. Wang G. Guo Q.. (2014). Overexpression of monoubiquitin improves photosynthesis in transgenic tobacco plants following high temperature stress. Plant Sci. Int. J. Exp. Plant Biol. 226, 92–100. 10.1016/j.plantsci.2014.03.006 25113454

[B156] Tian M. Lou L. Liu L. Yu F. Zhao Q. Zhang H.. (2015). The RING finger E3 ligase STRF1 is involved in membrane trafficking and modulates salt-stress response in Arabidopsis thaliana. Plant J. 82, 81–92. 10.1111/tpj.12797 25704231

[B157] Tuteja N. Ahmad P. Panda B. B. Tuteja R. (2009). Genotoxic stress in plants: shedding light on DNA damage, repair and DNA repair helicases. Mutat. Res. 681, 134–149. 10.1016/j.mrrev.2008.06.004 18652913

[B158] Ueda M. Matsui K. Ishiguro S. Sano R. Wada T. Paponov I.. (2004). The HALTED ROOT gene encoding the 26S proteasome subunit RPT2a is essential for the maintenance of Arabidopsis meristems. Development 131, 2101–2111. 10.1242/dev.01096 15073153

[B159] Ueda M. Seki M. (2020). Histone modifications form epigenetic regulatory networks to regulate abiotic stress response. Plant Physiol. 182, 15–26. 10.1104/pp.19.00988 31685643 PMC6945856

[B160] Uno Y. Furihata T. Abe H. Yoshida R. Shinozaki K. Yamaguchi-Shinozaki K. (2000). Arabidopsis basic leucine zipper transcription factors involved in an abscisic acid-dependent signal transduction pathway under drought and high-salinity conditions. *Proc. Natl. Acad. Sci*. U. S. A. 97, 11632–11637. 10.1073/pnas.190309197 11005831 PMC17252

[B161] van Veen H. Mustroph A. Barding G. A. Vergeer-van Eijk M. Welschen-Evertman R. A. Pedersen O.. (2013). Two Rumex species from contrasting hydrological niches regulate flooding tolerance through distinct mechanisms. Plant Cell 25, 4691–707. 10.1105/tpc.113.119016 24285788 PMC3875744

[B162] Vierstra R. D. (2009). The ubiquitin−26S proteasome system at the nexus of plant biology. Nat. Rev. Mol. Cell Biol. 10, 385–397. 10.1038/nrm2688 19424292

[B163] Walsh C. K. Sadanandom A. (2014). Ubiquitin chain topology in plant cell signaling: a new facet to an evergreen story. Front. Plant Sci. 5, 122. 10.3389/fpls.2014.00122 24744767 PMC3978257

[B164] Wan X. Mo A. Liu S. Yang L. Li L. (2011). Constitutive expression of a peanut ubiquitin-conjugating enzyme gene in Arabidopsis confers improved water-stress tolerance through regulation of stress-responsive gene expression. J. Biosci. Bioeng. 111, 478–484. 10.1016/j.jbiosc.2010.11.021 21193345

[B165] Wang F. Zhu D. Huang X. Li S. Gong Y. Yao Q.. (2009a). Biochemical insights on degradation of Arabidopsis DELLA proteins gained from a cell-free assay system. The Plant Cell 21, 2378–2390. 10.1105/tpc.108.065433 19717618 PMC2751948

[B166] Wang S. Kurepa J. Smalle J. A. (2009b). The Arabidopsis 26S proteasome subunit RPN1a is required for optimal plant growth and stress responses. Plant Cell Physiol. 50, 1721–1725. 10.1093/pcp/pcp105 19605416

[B167] Weissman A. M. (2001). Themes and variations on ubiquitylation. Nat. Rev. Mol. Cell Biol. 2, 169–178. 10.1038/35056563 11265246

[B168] Welchman R. L. Gordon C. Mayer R. J. (2005). Ubiquitin and ubiquitin-like proteins as multifunctional signals. Nat. Rev. Mol. Cell Biol. 6, 599–609. 10.1038/nrm1700 16064136

[B169] Weng M. Yang Y. Feng H. Pan Z. Shen W. H. Zhu Y.. (2014). Histone chaperone ASF1 is involved in gene transcription activation in response to heat stress in Arabidopsis thaliana. Plant Cell Environ. 37, 2128–2138. 10.1111/pce.12299 24548003

[B170] Wu G. Xu G. Schulman B. A. Jeffrey P. D. Harper J. W. Pavletich N. P. (2003). Structure of a β-TrCP1-Skp1-β-catenin complex. Mol. Cell 11, 1445–1456. 10.1016/S1097-2765(03)00234-X 12820959

[B171] Wu Q. Zhang X. Peirats-Llobet M. Belda-Palazon B. Wang X. Cui S.. (2016). Ubiquitin ligases RGLG1 and RGLG5 regulate abscisic acid signaling by controlling the turnover of phosphatase PP2CA. Plant Cell 28, 2178–2196. 10.1105/tpc.16.00364 27577789 PMC5059804

[B172] Xu M. Zhang X. Yu J. Guo Z. Li Y. Song X.. (2021). Proteome-wide analysis of lysine 2-hydroxyisobutyrylation in *Aspergillus niger* in peanuts. Front. Microbiol. 12, 719337. 10.3389/fmicb.2021.719337 34489910 PMC8418202

[B173] Xue H. Zhang Q. Wang P. Cao B. Jia C. Cheng B.. (2022). qPTMplants: an integrative database of quantitative post-translational modifications in plants. Nucleic Acids Res. 50, D1491–D1499. 10.1093/nar/gkab945 34718741 PMC8728288

[B174] Yanagawa Y. Komatsu S. (2012). Ubiquitin/proteasome-mediated proteolysis is involved in the response to flooding stress in soybean roots, independent of oxygen limitation. Plant Sci. 185–186, 250–258. 10.1016/j.plantsci.2011.11.014 22325888

[B175] Yang R. Wang T. Shi W. Li S. Liu Z. Wang J.. (2020). E3 ubiquitin ligase ATL61 acts as a positive regulator in abscisic acid mediated drought response in Arabidopsis. Biochem. Biophys. Res. Commun. 528, 292–298. 10.1016/j.bbrc.2020.05.067 32499110

[B176] Ye Y. Rape M. (2009). Building ubiquitin chains: E2 enzymes at work. Nat. Rev. Mol. Cell Biol. 10, 755–764. 10.1038/nrm2780 19851334 PMC3107738

[B177] Yee D. Goring D. R. (2009). The diversity of plant U-box E3 ubiquitin ligases: from upstream activators to downstream target substrates. J. Exp. Bot. 60, 1109–1121. 10.1093/jxb/ern369 19196749

[B178] Yu F. Wu Y. Xie Q. (2016). Ubiquitin-proteasome system in ABA signaling: from perception to action. Mol. Plant 9, 21–33. 10.1016/j.molp.2015.09.015 26455462

[B179] Zhan E. Zhou H. Li S. Liu L. Tan T. Lin H. (2018). OTS1-dependent deSUMOylation increases tolerance to high copper levels in Arabidopsis. J. Integr. Plant Biol. 60, 310–322. 10.1111/jipb.12618 29205850

[B180] Zhang Y. Lai X. Yang S. Ren H. Yuan J. Jin H.. (2021). Functional analysis of tomato CHIP ubiquitin E3 ligase in heat tolerance. Sci. Rep. 11, 1713. 10.1038/s41598-021-81372-8 33462308 PMC7814054

[B181] Zhang Y. Xu W. Li Z. Deng X. W. Wu W. Xue Y. (2008). F-box protein DOR functions as a novel inhibitory factor for abscisic acid-induced stomatal closure under drought stress in Arabidopsis. Plant Physiol. 148, 2121–2133. 10.1104/pp.108.126912 18835996 PMC2593669

[B182] Zhang Y. Yang C. Li Y. Zheng N. Chen H. Zhao Q.. (2007). SDIR1 is a RING finger E3 ligase that positively regulates stress-responsive abscisic acid signaling in Arabidopsis. Plant Cell 19, 1912–29. 10.1105/tpc.106.048488 17573536 PMC1955734

[B183] Zhang Y.-Y. Xie Q. (2007). Ubiquitination in abscisic acid-related pathway. J. Integr. Plant Biol. 49, 87–93. 10.1111/j.1744-7909.2006.00417.x

[B184] Zheng N. Shabek N. (2017). Ubiquitin ligases: structure, function, and regulation. Annu. Rev. Biochem. 86, 129–157. 10.1146/annurev-biochem-060815-014922 28375744

[B185] Zhou G.-A. Chang R.-Z. Qiu L.-J. (2010). Overexpression of soybean ubiquitin-conjugating enzyme gene GmUBC2 confers enhanced drought and salt tolerance through modulating abiotic stress-responsive gene expression in Arabidopsis. Plant Mol. Biol. 72, 357–367. 10.1007/s11103-009-9575-x 19941154 PMC2816239

[B186] Zhou M. Chen H. Wei D. Ma H. Lin J. (2017). Arabidopsis CBF3 and DELLAs positively regulate each other in response to low temperature. Sci. Rep. 7, 39819. 10.1038/srep39819 28051152 PMC5209670

[B187] Zhu J. Dong C.-H. Zhu J.-K. (2007). Interplay between cold-responsive gene regulation, metabolism and RNA processing during plant cold acclimation. Curr. Opin. Plant Biol. 10, 290–295. 10.1016/j.pbi.2007.04.010 17468037

